# Supplementary material for: Amine Landscaping to Maximize Protein-Dye Fluorescence and Ultrastable Protein-Ligand Interaction
Source: Cell Chem Biol. 2017 Aug 17;24(8):1040–1047.e4. doi: 10.1016/j.chembiol.2017.06.015 (PMC5563079; doi:10.1016/j.chembiol.2017.06.015)
Supplement: Document S1. Figures S1–S5 and Table S1 [file mmc1.pdf]

Cell Chemical Biology, Volume *24*

Supplemental Information

Amine Landscaping to Maximize Protein-Dye Fluorescence and Ultrastable Protein-Ligand Interaction

Michael T. Jacobsen, Michael Fairhead, Per Fogelstrand, and Mark Howarth

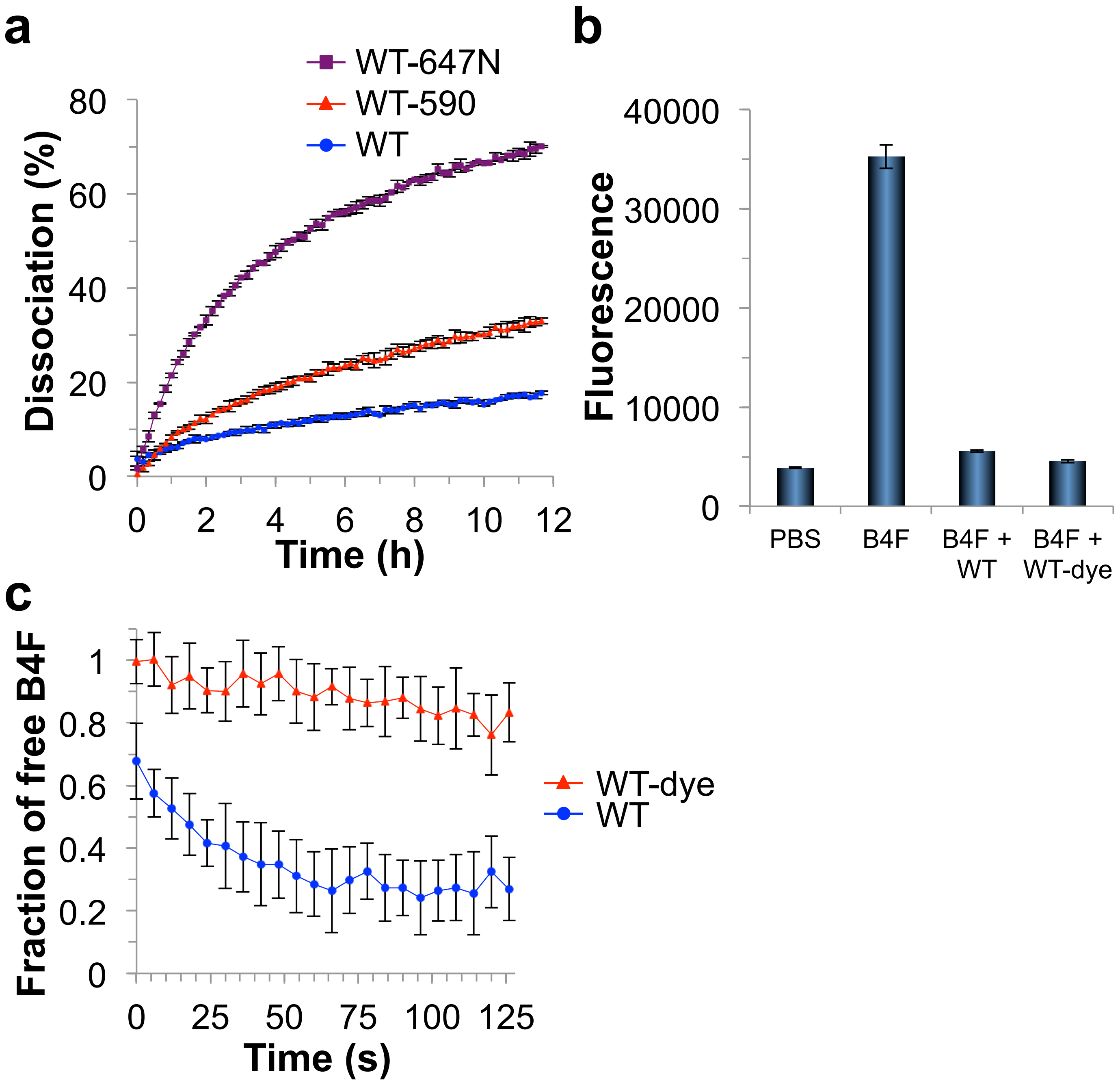

Fig. S1 (related to Fig. 1). Dye labeling of streptavidin impaired ligand binding. (a) Increased dissociation rate after labeling streptavidin with alternative dyes. Biotin-4-fluorescein (B4F) dissociation rates for WT streptavidin unmodified or after conjugation with Atto647N-NHS or Atto590-NHS (mean of triplicate ± 1 s.d.). (b) Streptavidin conjugation with dye had little effect on initial B4F quenching. Raw fluorescence values (arbitrary units) in the B4F dissociation rate assay for WT streptavidin and 635P-NHS-labeled WT streptavidin, immediately before addition of excess biotin, from Fig. 1a (mean ± 1 s.d., n=3). Controls with PBS alone or B4F alone are included. (c) Labeling slowed ligand binding by streptavidin. Fraction of free B4F after incubation with WT streptavidin or 635P-NHS-labeled streptavidinin. Compared to Fig. 1b, the data are shown for a longer time and with a linear y-axis (mean ± 1 s.d., n=9).

a

```
Streptavidin          ---MRKIVVAAIAVSLTTVSITASASADPSKD-----SKAQVSAAEAGITGTWYNQLGSTFIVTAG-A-D
Avidin                -----------MVHATSPLLLLLLLSLALVAPG--------LSARKCSLTGKWTNDLGSNMTIGAVNS-R
Neutralite Avidin     -----------MVHATSPLLLLLLLSLALVAPG--------LSAAICSLTGEWTNDLGSIMTIGAVNS-N
Bradavidin I          ------------MRHFNGMLLAMIASTSLIGP---------LPAYAQSVNWTWTNQYGSTLAITSFNSNT
Tamavidin 1           ----------------------------------------MKDVQSLLTGTWYNELGSTMNLTAN-K-D
Tamavidin 2           ----------------------------------------MSDVQSSLTGTWYNELNSKMELTAN-K-D
Xenavidin             ------------MNALTLLAVALALYGCICTE-----------AQKCNLQGQWRNKLGSNLIIESV-SQN
S. virginiae          ---MRKIVVAAIAVSLTTISITASASADPSKD-----SKAQVSAAEAGITGTWYNQLGSTFIVTAG-A-D
S. katrae             ---MRKIVVAAIAVSLTTVGIAANASADPAKD-----SKAQVAAVDAGITGTWYNQLGSTFTVTAN-T-D
S. flavotricini       MTRVRQTLVALCALSLTFVTVSASASADTRATVSQTAESGSVTESRPGILGTWYNQLGSVMVVTRA-A-N
S. hygroscopicus      ----------------MTVEVTVRASREKIMP----------------IDGEWYNEFGSQVRLESA-P-E

Streptavidin          GALTGTYESAVGNAESRYVLTGRYDSAPATDG-SGTALGWTVAWKNNYRNAHSATTWSGQ--YVGGAEAR
Avidin                GEFTGTYITAVTATSNEIKESPLHGTQNTINKRTQPTFGFTVNWK----FSESTTVFTGQCFIDRNGKEV
Neutralite Avidin     GEFTGTYITAVTATSNEIKESPLHGTQNTINKATQPTFGFTVNWK----FSESTTVFTGQCFIDRNGKEV
Bradavidin I          GAITGTYTNNAANSCDEGKPQGVTGWLAYGNT--GTAISFSVNFL----GCGSTTVWTGQ----LNNATG
Tamavidin 1           GSLTGTYHSNVGEVPPTYHLSGRYNLQPPSGQ--GVTLGWAVSFENTSANVHSVSTWSGQ--YFSEPAEV
Tamavidin 2           GTLTGKYLSKVGDVYVPYPLSGRYNLQPPAGQ--GVALGWAVSWEN--SKIHSATTWSGQ--FFSESSPV
Xenavidin             GEFTGTYFTSVSLTNSTIRISPLTGYQKLTEK---PTFGFTVHWA----FSDSITVWTGQCFLNEKGEEI
S. virginiae          GALTGTYESAVGNAESRYVLTGRYDSTPATDG-SGTALGWSVAWKNNYRNAHSATTWSGQ--YVGGAEAR
S. katrae             GSLTGTYESAVGNAESRYVVTGRYDTAAAT-G-TGTALGWTVAWKNDFRNAHSATTWSGQ--YVGGADAK
S. flavotricini       GGFTGTYESAVGNAEKRYTMTGRYDSAPAD-G-TGTAVGWTVAYRNAHRNAHSVATWSGQ--YVGGSQER
S. hygroscopicus      GTVSGEYVTAVGHAPGTYLLTGRHDG-PTSPG-NGIALGWTVAWRNEHGNLGSVTSWSGL---FLEGPER

Streptavidin          INTQWLLTSGT-TEANAWKSTLVGHDTFTKVKPSAASIDAAKKAGVNNGNPLDAVQQ
Avidin                LKTMWLLRSSVNDIGDDWKATRVGINIFTRLRTQKE---------------------
Neutralite Avidin     LKTMWLLRSSVNDIGDDWKATRVGINIFTALATQKE---------------------
Bradavidin I          FQGLWYLSLAE---AVAWNGISAGADTFTFSSGDKALLTKSGVDLKAGSEKLSNTKK
Tamavidin 1           ILTQWLLSRSS-EREDLWQSTHVGHDEFSKTKPTKEKIAQAQLL--RRGLKFE----
Tamavidin 2           ILTQWLLSSST-ARGDVWESTLVGNDSFTKTAPTEQQIAHAQLH--CRAPRLK----
Xenavidin             LHTMWLLRSSQEKEQDNWTGTRVGGNTFTRLSKKKIRKE------------------
S. virginiae          INTQWLLTSGT-TEANAWKSTLVGHDTFTKVKPSAASIDAAKKAGINNGNPLDAPQQ
S. katrae             INTQWLLTSGT-TAADSWKSTLVGHDTFTKVKPSAASIDAAKKAGVNNGNPLDAVQQ
S. flavotricini       IVTQWLLSYGT-TPADQWKSTVLGHDEFTRVKPSAADIEKARRLGVTTANPQASEGE
S. hygroscopicus      ILTTWLLSRST-ATAEAWESSVVGQDVFTRQKPTSEDVDRALRSGRPVSHPMPS---
```

b

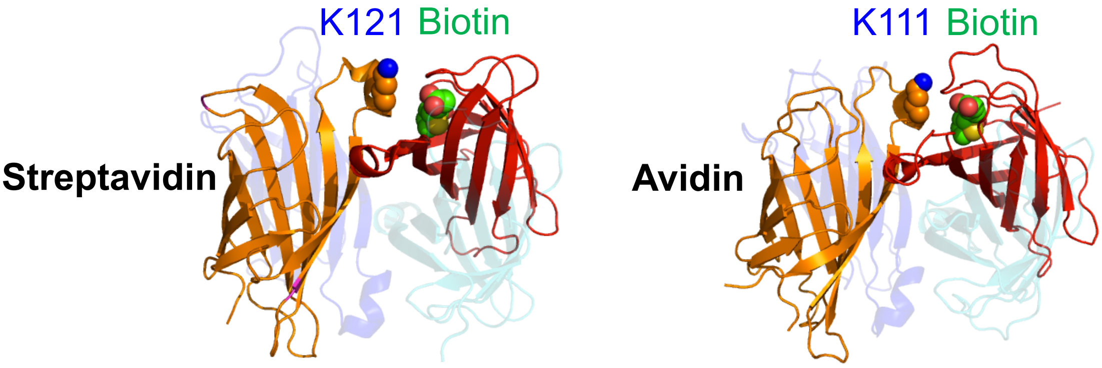

Fig. S2 (related to Fig. 2). Conservation of biotin-proximal lysine in the avidin family. (a) Amino acid sequence alignment of streptavidin-related tetramers, including well-characterized avidins (streptavidin, avidin, neutralite avidin, bradavidin I, tamavidin 1 & 2, and xenavidin) plus four other variants from the *Streptomyces* genus. Fully conserved residues are colored in red. The equivalent residue to streptavidin's K121 is boxed and is colored blue if Lys is conserved. Dimeric avidins such as rhizavidin were not included because they do not possess the relevant protein interface. (b) Structural conservation of the proximity between Lys in subunit 1 (orange) and biotin in subunit 2 (red) of streptavidin (PDB 3RY2) and avidin (PDB 1AVD).

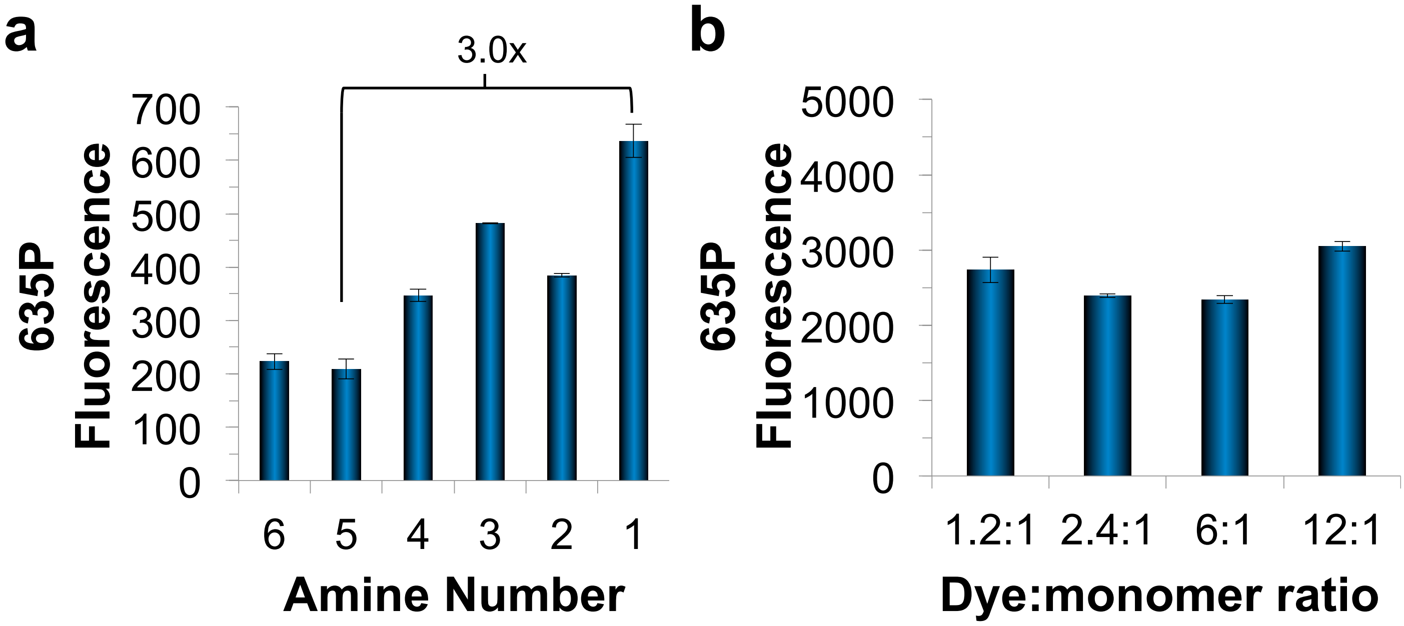

Fig. S3 (related to Fig. 3). Brightness of streptavidin-dye conjugates. (a) The relationship between fluorescence brightness and amine number was maintained at low protein concentration. Fluorescent brightness (in arbitrary units) of amine mutation series conjugated with 635P-NHS, tested with 0.1 μM protein (mean of triplicate ± 1 s.d.). (b) Fluorescent brightness of WT streptavidin after incubation at the indicated dye:monomer ratios with 635P-NHS. Measurement of fluorescence (in arbitrary units) for protein-dye conjugates (mean of triplicate ±1 s.d.).

a

WT	MAEAGITGTWYNQLGSTFIVTAGADGALTGTYESAVGNAE
Flavidin	MAEAGITGTWYNQLGSTFIVTAGADGALTGTYESAVGNAE
WT	SRYVLTGRYDSAPATDGSGTALGWTVAWKNNYRNAHSATT
Flavidin	SRYVLTGRYDSAPATDGSGTALGWTVAW**R**NNYRNAHSATT
WT	WSGQYVGGAEARINTQWLLTSGTTEANAWKSTLVGHDTFT
Flavidin	WSGQYVGGAEARINTQWLLTSGTTEANAW**R**STLVGHDTFT
WT	KVKPSAASEEEEEE
Flavidin	**R**V**R**PSAASEEEEEE

b

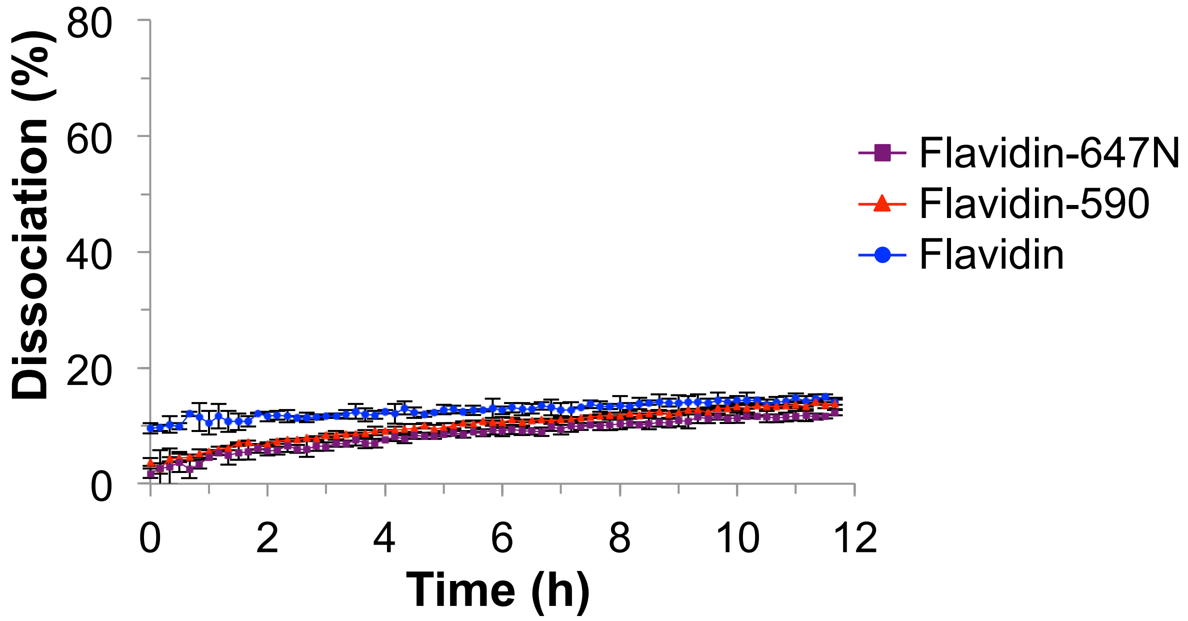

Fig. S4 (related to Fig. 4). Flavidin sequence and ligand dissociation. (a) Amino acid sequences of WT core streptavidin and Flavidin. Mutated residues in Flavidin are marked in red. There is a hexaglutamate tag at each C-terminus for purification. (b) Flavidin ligand dissociation rate was not affected by labeling with alternative dyes. Biotin-4-fluorescein dissociation rates for Flavidin unmodified or after conjugation with Atto647N-NHS or Atto590-NHS (mean of triplicate ± 1 s.d.).

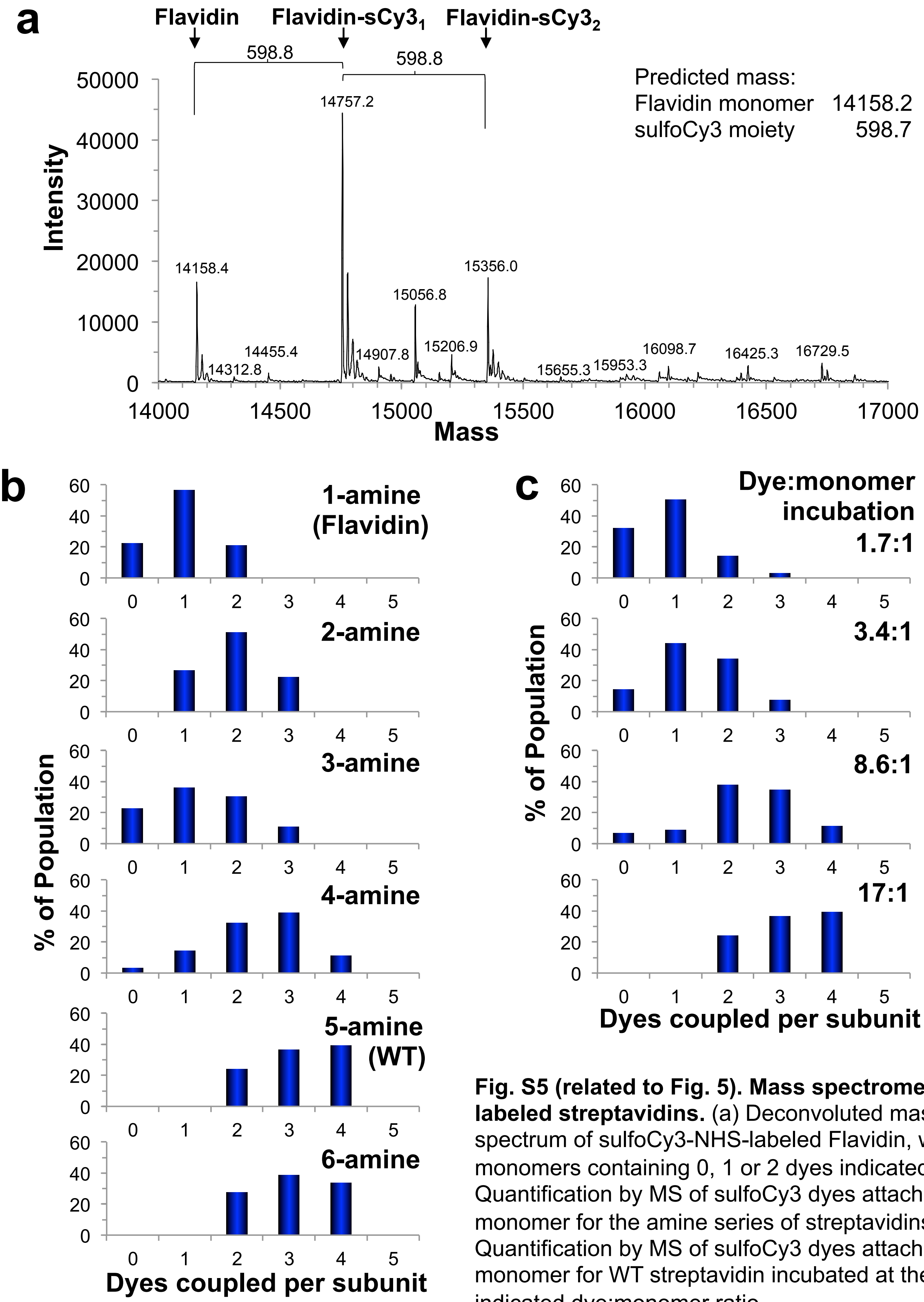

Fig. S5 (related to Fig. 5). Mass spectrometry of labeled streptavidins. (a) Deconvoluted mass spectrum of sulfoCy3-NHS-labeled Flavidin, with monomers containing 0, 1 or 2 dyes indicated. (b) Quantification by MS of sulfoCy3 dyes attached per monomer for the amine series of streptavidins. (c) Quantification by MS of sulfoCy3 dyes attached per monomer for WT streptavidin incubated at the indicated dye:monomer ratio.

Table S1: Oligonucleotides used in this work. Related to STAR Methods section.

Purpose	Oligonucleotide	Oligonucleotide Sequence (5' to 3')
Introduce K121R mutation by QuikChange	SA-K121R-For	GCTAACGCGTGGCGATCCACCCTGGTTGG
…	SA-K121R-Rev	CCAACCAGGGTGGATCGCCACGCGTTAGC
Introduce K121E mutation by QuikChange	SA-K121E-For	GCTAACGCGTGGGAATCCACCCTGGTTGG
…	SA-K121E-Rev	CCAACCAGGGTGGATTCCCACGCGTTAGC
Introduce K121Q mutation by QuikChange	SA-K121Q-For	GCTAACGCGTGGCAATCCACCCTGGTTGG
…	SA-K121Q-Rev	CCAACCAGGGTGGATTGCCACGCGTTAGC
Introduce K121A mutation by QuikChange	SA-K121A-For	GCTAACGCGTGGGCATCCACCCTGGTTGG
…	SA-K121A-Rev	CCAACCAGGGTGGATGCCCACGCGTTAGC
Introduce N82K mutation by Gibson Assembly	SA-N82K-Gib-For	CCGTTGCTTGGAAAAACAAATACCGTAACG CTCACTCCGCTACCACC
…	AmpB	GATCGTTGTCAGAAGTAAGTTGGCC
…	SA-N82K-Gib-Rev	GTTTTTCCAAGCAACGGTCCAACC
…	AmpA	GGCCAACTTACTTCTGACAACGATC
Introduce R103K mutation by Gibson Assembly	SA-R103K-Gib-For	CAGTACGTTGGTGGTGCTGAAGCTAAAATC AACACCCAGTGGTTGTTGACC
…	AmpB	GATCGTTGTCAGAAGTAAGTTGGCC
…	SA-R103K-Giv-Rev	ACCACCAACGTACTGGCCAGACC
…	AmpA	GGCCAACTTACTTCTGACAACGATC
Introduce K80R mutation by Gibson Assembly	SA-K80R-Gib-For	TCTGGGTTGGACCGTTGCTTGGCGCAACAA CTACCGTAACGCTCAC
…	AmpB	GATCGTTGTCAGAAGTAAGTTGGCC
…	SA-K80R-Gib-Rev	CCAAGCAACGGTCCAACCCAGAG
…	AmpA	GGCCAACTTACTTCTGACAACGATC
Introduce K132R and K134R mutations by Gibson Assembly	SA-K132RK134R-For	GGTCACGACACCTTCACCCGTGTTCGTCCG TCCGCTGCTTCCG
…	AmpB	GATCGTTGTCAGAAGTAAGTTGGCC
…	SA-K132RK134R-Rev	GGTGAAGGTGTCGTGACCAACC
…	AmpA	GGCCAACTTACTTCTGACAACGATC
