# Supplementary material for: Amine Landscaping to Maximize Protein-Dye Fluorescence and Ultrastable Protein-Ligand Interaction
Source: Cell Chem Biol. 2017 Aug 17;24(8):1040–1047.e4. doi: 10.1016/j.chembiol.2017.06.015 (PMC5563079; doi:10.1016/j.chembiol.2017.06.015)
Supplement: Document S2. Article plus Supplemental Information [file mmc2.pdf]

Resource

Cell Chemical Biology

Amine Landscaping to Maximize Protein-Dye Fluorescence and Ultrastable Protein-Ligand Interaction

Graphical Abstract

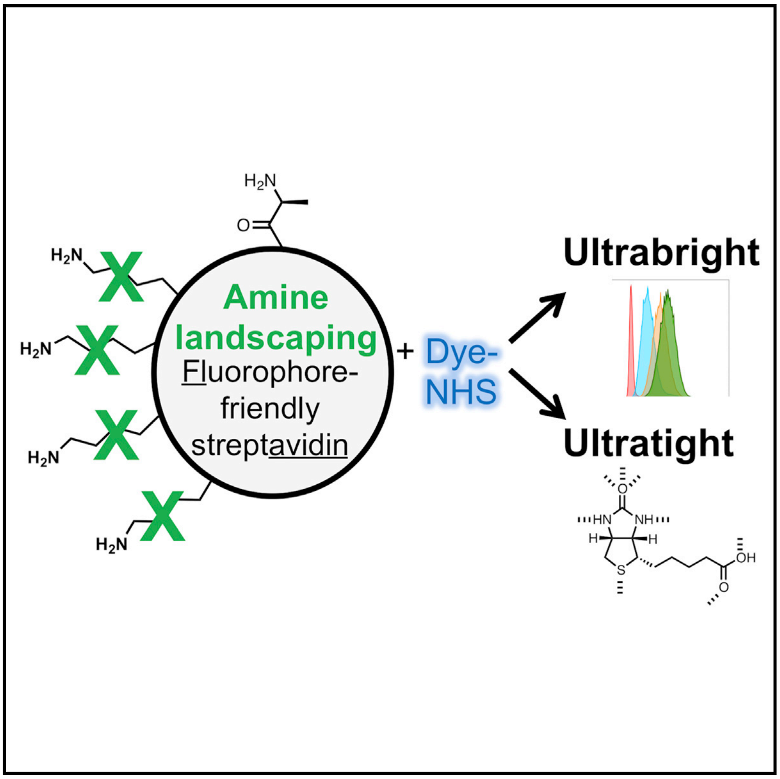

Highlights

- Labeling of streptavidin with small-molecule dyes impairs ligand binding
- K121R mutation rescues ligand stability after dye labeling
- Landscaping of protein amines optimizes brightness
- Fluorophore-friendly streptavidin improves imaging specificity and sensitivity

Authors

Michael T. Jacobsen, Michael Fairhead, Per Fogelstrand, Mark Howarth

Correspondence

mark.howarth@bioch.ox.ac.uk

In Brief

Chemical coupling of dyes frequently damages protein function. Jacobsen et al. explored this challenge using the widespread tool streptavidin. Through an amine-landscaping strategy, they produced "Fluorophore-friendly streptavidin" which combines extreme ligand-binding stability with brighter and more specific cellular imaging performance.

Jacobsen et al., 2017, Cell Chemical Biology *24*, 1040–1047
August 17, 2017 © 2017 The Author(s). Published by Elsevier Ltd.
http://dx.doi.org/10.1016/j.chembiol.2017.06.015

CellPress

Cell Chemical Biology
Resource

Amine Landscaping to Maximize Protein-Dye Fluorescence and Ultrastable Protein-Ligand Interaction

Michael T. Jacobsen,[1] Michael Fairhead,[1] Per Fogelstrand,[2] and Mark Howarth[1,3,*]
[1]Department of Biochemistry, University of Oxford, South Parks Road, Oxford OX1 3QU, UK
[2]Wallenberg Laboratory, Department of Molecular and Clinical Medicine, Institute of Medicine, University of Gothenburg, Gothenburg, Sweden
[3]Lead Contact
*Correspondence: mark.howarth@bioch.ox.ac.uk
http://dx.doi.org/10.1016/j.chembiol.2017.06.015

SUMMARY

Chemical modification of proteins provides great opportunities to control and visualize living systems. The most common way to modify proteins is reaction of their abundant amines with N-hydroxysuccinimide (NHS) esters. Here we explore the impact of amine number and positioning on protein-conjugate behavior using streptavidin-biotin, a central research tool. Dye-NHS modification of streptavidin severely damaged ligand binding, necessitating development of a new streptavidin-retaining ultrastable binding after labeling. Exploring the ideal level of dye modification, we engineered a panel bearing 1–6 amines per subunit: "amine landscaping." Surprisingly, brightness increased as amine number decreased, revealing extensive quenching following conventional labeling. We ultimately selected Flavidin (fluorophore-friendly streptavidin), combining ultrastable ligand binding with increased brightness after conjugation. Flavidin enhanced fluorescent imaging, allowing more sensitive and specific cell labeling in tissues. Flavidin should have wide application in molecular detection, providing a general insight into how to optimize simultaneously the behavior of the biomolecule and the chemical probe.

INTRODUCTION

Chemically derivatizing proteins allows huge expansion of their functional and translational potential. Such modifications can alter catalytic activity or circulation time, allow delivery of drugs and radioisotopes, bridge distinct biomolecules, or allow imaging *in vitro* or in living organisms (Agarwal and Bertozzi, 2015; Liu et al., 2015; Stephanopoulos and Francis, 2011). Lysine is one of the most abundant residues at the surface of proteins and so amine-based conjugation allows multiple modifications per protein. For example, there are ~80 Lys per immunoglobulin G antibody (Wang et al., 2005). One of the most common chemical modifications of proteins is fluorescent labeling, particularly for microscopy, diagnostics, and flow cytometry (Sano et al., 1998). The vast majority of fluorescent dyes are provided as N-hydroxysuccinimide (NHS) esters or sulfo-NHS esters (hereafter grouped as NHS); NHS esters react with amine groups on proteins (N-terminal α-amine or Lys ε-amine) to form a stable amide bond (Bragg and Hou, 1975). After NHS dyes, the second most available reactive dyes are maleimides. The precision of maleimide labeling of rare surface Cys is indeed very useful. However, surface Cys can undergo competing disulfide bond formation and, in the case of tetramers such as streptavidin, multimerization through disulfides can quickly lead to precipitation. Furthermore, maleimide conjugates can re-arrange, hydrolyze, or exchange in the presence of other thiols (Shinmi et al., 2016). There is a wide literature on the use of NHS-dye conjugates, with some examples where labeling interferes with binding properties and examples of excess dye labeling reducing overall fluorescence (Vira et al., 2010; Zanetti-Domingues et al., 2013). However, in such systems, amine modification sites have rarely been changed, which would enable precise control of the potential reaction sites and optimization of molecular properties, e.g., ligand-binding kinetics, protein stability, and fluorescent brightness.

We first set out to explore whether dye modification had an effect on the ligand-binding properties of streptavidin. Streptavidin-biotin is one of the strongest and most widely used protein-ligand interactions (Chilkoti et al., 1995a; Laitinen et al., 2006; Sano et al., 1996). The binding of biotin by streptavidin or avidin is a model of molecular recognition, achieving exceptional stability despite the small contact surface area (Houk et al., 2003; Kuntz et al., 1999). We discovered that dye modification resulted in a significant impairment to biotin-conjugate binding, which we overcame by structure-based engineering. Using a novel amine-landscaping strategy, we then established how to simultaneously maintain ultrastable ligand binding and achieve maximal fluorescent brightness of the protein-dye conjugate.

RESULTS

Dye Labeling Impaired Ligand Binding

Wild-type (WT) core streptavidin (Sano et al., 1995) was labeled using Abberior STAR 635P NHS carbonate. The 635P dye was chosen because of its excellent photophysical characteristics (extinction coefficient, quantum yield, and photostability) and

 © 2017 The Author(s). Published by Elsevier Ltd.
This is an open access article under the CC BY license (http://creativecommons.org/licenses/by/4.0/).

because its absorption and emission spectra are well separated from fluorescein (Wu et al., 2015). We removed unreacted dye by gel filtration and three rounds of dialysis. We used biotin-4-fluorescein as an efficient readout of ligand binding with streptavidin. Biotin-4-fluorescein fluorescence is quenched by 90% upon streptavidin binding. Therefore, biotin-4-fluorescein's off rate, induced by adding excess free biotin, can be continuously monitored from the recovery of fluorescence upon dissociation from streptavidin's binding pocket (Kada et al., 1999). Dye labeling of WT streptavidin produced a dramatic increase in biotin-4-fluorescein dissociation rate (Figure 1A). After 10 hr, WT streptavidin had 13% ± 1% dissociation, whereas more than half of the dye-labeled protein had lost its ligand (52% ± 0.7%, mean of triplicate ± 1 SD) (Figure 1A). We also observed increased dissociation rates after labeling WT streptavidin with two other commercially available dyes with good fluorescence characteristics (Cordes et al., 2011) (Atto647N-NHS and Atto590-NHS; Figure S1A). Initial quenching of biotin-4-fluorescein, prior to biotin addition, was similarly efficient in both WT and WT-dye samples (Figure S1B).

To study the effects of dye labeling on streptavidin's rate of ligand *association*, we mixed streptavidin with biotin-4-fluorescein and followed the rate of quenching of the fluorescence of biotin-4-fluorescein. Labeling of streptavidin with 635P reduced the association rate by an order of magnitude (Figure 1B): 5.7 ± 0.3 × 10^7 M^{-1} s^{-1} for WT streptavidin and 5.7 ± 2.2 × 10^6 M^{-1} s^{-1} after 635P labeling. The fraction of free B4F plotted against time reinforced the severe impact of dye labeling on streptavidin association rate (Figure S1C).

To further probe the susceptibility of WT streptavidin to labeling-induced impairment, we tuned the dye:protein ratio in the labeling reaction. The biotin-conjugate off rate was increased at even the lowest dye:protein monomer ratio of 1.2:1 (Figure 1C). Thus, biotin-conjugate binding by WT streptavidin is highly sensitive to dye labeling: it is not feasible to try to preserve ultrastable biotin-conjugate binding through gentle dye-NHS labeling.

Interaction at K121 Is the Key to Dye's Effects on Ligand Binding

Based on analysis of the crystal structure of streptavidin (Freitag et al., 1999), the amine in closest proximity to the biotin-binding site comes from K121 of the neighboring subunit at the 1,2 subunit interface (8.7 Å from ε-nitrogen to biotin's carboxylate carbon) (Figure 2A) (Ramachandiran et al., 2007). We measured all amine-to-biotin distances in a high-resolution crystal structure (PDB: 3RY2) and found that this distance was the only Lys-to-biotin distance less than 10 Å. Consistent with this analysis, it is well established that W120 from the 1,2 subunit interface contributes an important interaction to biotin binding in tetrameric avidins (Chilkoti et al., 1995b; Sano and Cantor, 1995). We hypothesized that NHS-dye labeling of K121 led to a steric clash between the dye and the biotin ligand in the neighboring subunit. Sequence alignment of avidin-family tetramers (Hytonen et al., 2005; Maatta et al., 2009; Marttila et al., 2000; Nordlund et al., 2005; Takakura et al., 2010; Taskinen et al., 2014a) showed Lys frequently found at this position (Figure S2A), including in avidin (Figure S2B). Thus, we created a series of mutations at K121 to remove the Lys ε-amine, and expressed these mutant proteins in *Escherichia coli*. Each streptavidin mutant

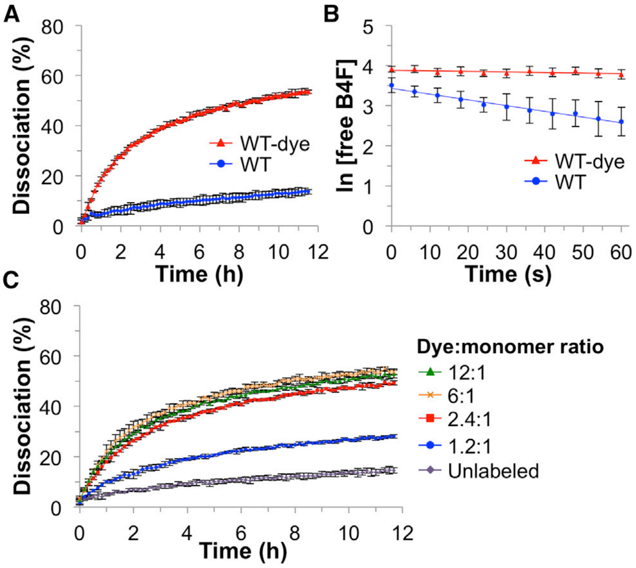

Figure 1. Labeling Impaired Ligand Binding by Streptavidin

(A) Biotin-4-fluorescein dissociation rate from unlabeled wild-type streptavidin (WT) or 635P-NHS-labeled streptavidin (WT-dye), determined from the increase in fluorescence after excess free biotin is added (mean of triplicate ± 1 SD).

(B) Biotin-4-fluorescein association rate to unlabeled wild-type streptavidin (WT) or 635P-NHS-labeled streptavidin (WT-dye), determined from the decrease in fluorescence upon mixing (mean ± 1 SD, n = 9).

(C) Biotin-4-fluorescein dissociation rate from WT streptavidin, unlabeled or labeled with varying 635P dye:protein monomer ratios (mean of triplicate ± 1 SD). See also Figure S1.

was well expressed, could be refolded from inclusion bodies, and formed a tetramer stable to SDS (Figure 2B). Comparing biotin ligand dissociation for each tetramer (without dye labeling of the protein), K121Q and K121E mutants had impaired biotin-conjugate binding, while the K121R and K121A mutants retained WT-like binding (Figure 2C). Since K121R possesses the same charge as WT at neutral pH and maintained good biotin-conjugate binding, we focused on this mutant. As hoped, after 635P dye labeling of K121R streptavidin, there was no loss in stability of biotin-conjugate binding (Figure 2D). K121R mutation also minimized the negative effect of dye labeling on ligand association (Figure 2E). The on rate for biotin-4-fluorescein binding to K121R streptavidin was 4.4 ± 0.7 × 10^7 M^{-1} s^{-1}, compared with 2.6 ± 0.2 × 10^7 M^{-1} s^{-1} after 635P labeling, a decrease upon labeling of approximately 2-fold rather than 10-fold for WT streptavidin.

Amine Landscaping of Streptavidin

To achieve optimal fluorescence brightness, we explored the importance of every Lys in streptavidin. WT core streptavidin possesses five amines per subunit (α-amine and four ε-amines at Lys). Each one of the four native Lys was mutated to Arg (Figure 3A). In addition, we tested the introduction of two new Lys via N82K and R103K mutations. N82 and R103 sites were chosen because of their high surface accessibility and because they are not adjacent to the biotin-binding site. Overall, we expressed streptavidin mutants ranging from one amine (just the α-amine) up to six amines per subunit (Figure 3A). All variants were well

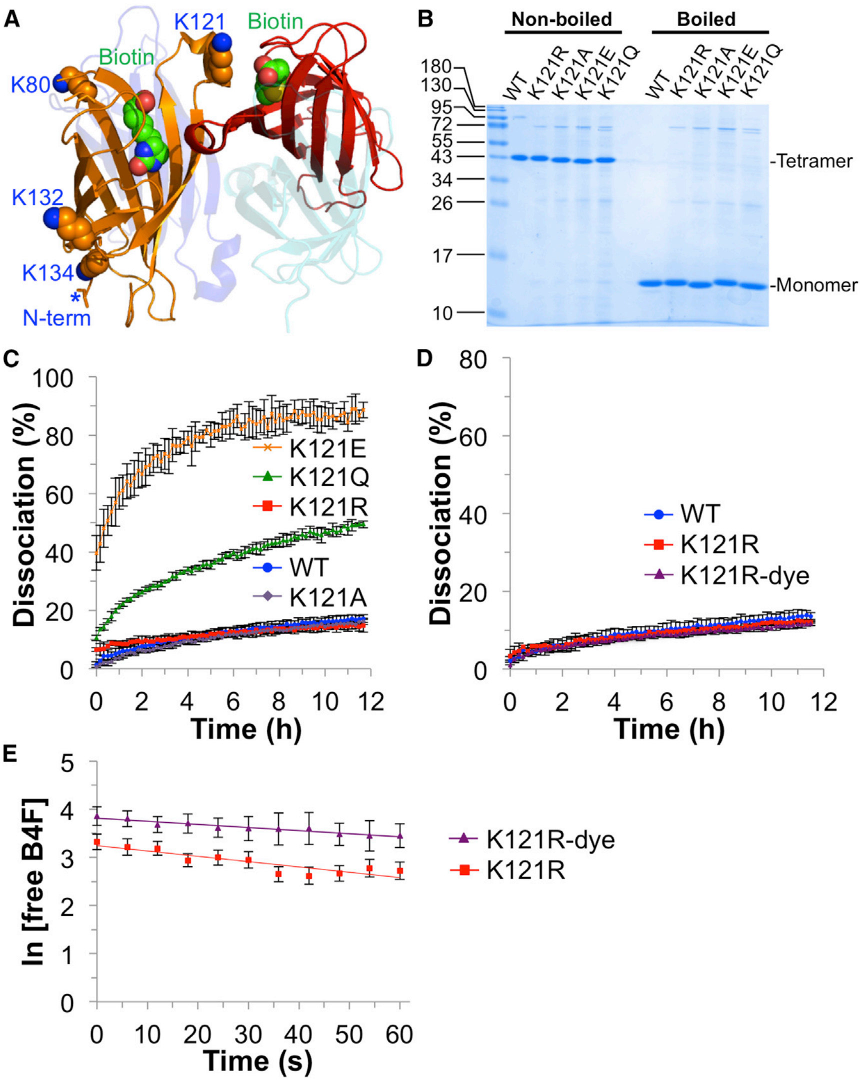

Figure 2. K121R Mutation Circumvented the Labeling Problem

(A) Amine positioning on WT streptavidin tetramer. Lys side chains on subunit 1 (orange) are shown in space-fill, with ε-amino nitrogens labeled in blue. Biotin with carbons in green is shown in space-fill for subunits 1 and 2 (red) (based on PDB: 3RY2). *Indicates the most N-terminal residue resolved in the crystal structure.

(B) Tetramer integrity of K121 streptavidin mutants, analyzed by SDS-PAGE with or without boiling and then Coomassie blue staining.

(C) Biotin-4-fluorescein dissociation rates for K121 mutants (mean of triplicate ± 1 SD).

(D) Biotin-4-fluorescein dissociation rates for K121R with or without 635P-NHS labeling, compared with WT (mean of triplicate ± 1 SD).

(E) Biotin-4-fluorescein association rates for K121R with or without 635P-NHS labeling (mean ± 1 SD, n = 9). See also Figure S2.

expressed in *E. coli* and refolded efficiently from inclusion bodies to the expected tetramers (Figure 3B).

Spectroscopic Characterization of the Amine-Landscape Series

The streptavidin series with one to six amines was labeled with 635P, normalized by protein concentration, and the absorption spectra were overlaid (Figure 3C). This analysis showed a correlation between the number of amines and the intensity of the left shoulder peak at 590 nm (hypsochromic to the principal peak at 637 nm). This hypsochromic peak is considered a marker of H-type dimer dye-dye interactions (Pauli et al., 2011; Schobel et al., 2000). We then measured the fluorescence brightness of the 635P-labeled series (Figure 3D). Interestingly, the degree of brightness inversely correlated with the number of amines in the protein. That is, for 635P-dye labeling of streptavidin, fewer amines led to greater brightness. We explored whether this effect was dependent on the interactions within the tetramer, rather than between tetramers, by re-measuring the brightness at 10-fold lower protein concentration. The same pattern of brightness was seen at 0.1 μM protein as at 1 μM protein (Figures 3D and S3A). It is natural to wonder whether simply using a very low dye:protein ratio when labeling WT streptavidin would achieve high fluorescent brightness. In fact, we found that WT streptavidin brightness was surprisingly insensitive to dye:protein ratio (Figure S3B).

We next labeled the streptavidin series with another popular fluorescent dye, sulfoCy3-NHS, and evaluated the absorbance spectra and fluorescent brightness. With sulfoCy3, we observed a similar correlation between amine number and the intensity of the left shoulder peak (hypsochromic peak at 514 nm; Figure 3E). The variant with the fewest amines showed the highest fluorescent brightness following sulfoCy3 labeling (Figure 3F).

Since the mutant with one amine showed the best fluorescent characteristics, we termed this variant Flavidin (fluorophore-friendly streptavidin) (sequence in Figure S4A) and further validated its behavior.

Biophysical Characterization of Flavidin

Many mutations to streptavidin impair its folding efficiency, biotin binding, or tetramer stability (Laitinen et al., 2006). We found that Flavidin was efficiently expressed and refolded from *E. coli* culture. Typical expression yields were 23 mg/L of culture for Flavidin, compared with 17 mg/L of culture for WT streptavidin. Flavidin showed good solubility: at least 100 μM in PBS. We characterized Flavidin tetramer stability by heating at various temperatures, followed by analysis via SDS-PAGE. Flavidin retained high thermostability similar to WT streptavidin (Chivers et al., 2010) (Figure 4A). Flavidin had an on rate for biotin-4-fluorescein of 5.1 ± 0.7 × 10^7 M^{-1} s^{-1}, while for Flavidin-635P the value was 2.1 ± 0.3 × 10^7 M^{-1} s^{-1} (Figure 4B)

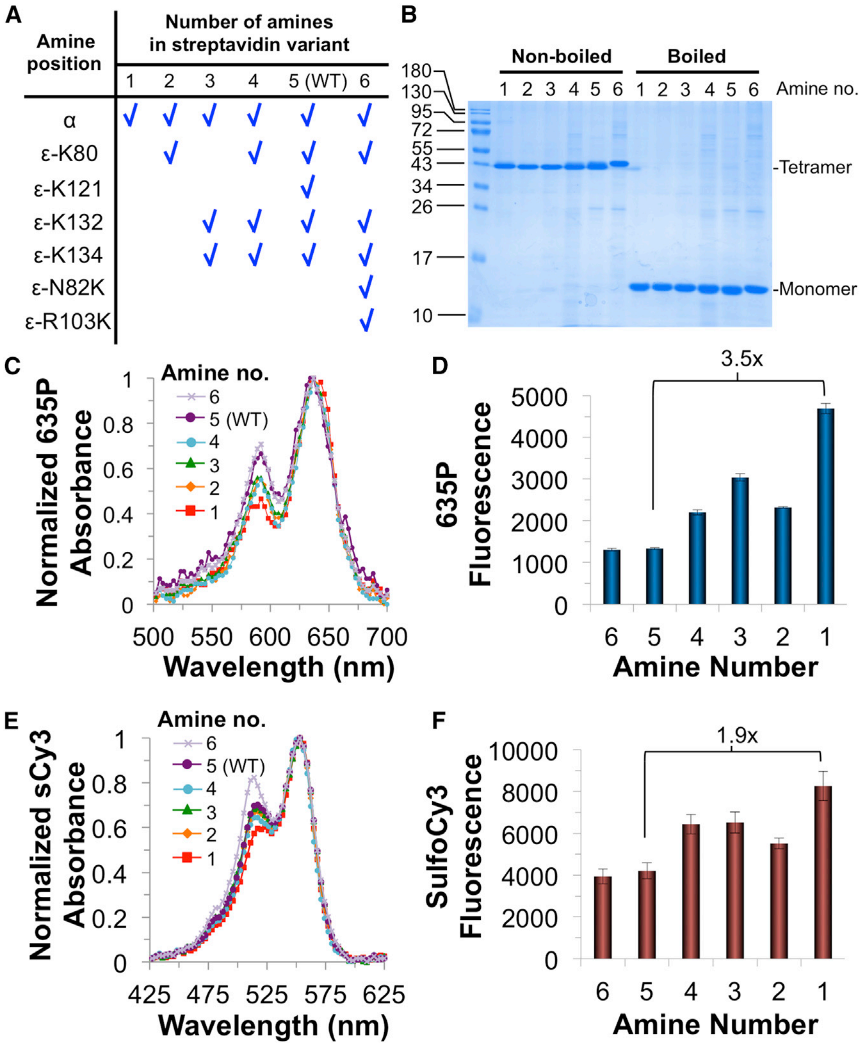

Figure 3. Amine Landscaping to Increase Streptavidin-Conjugate Brightness

(A) Amine composition of the streptavidin mutation series, one amine: K80R, K121R, K132R, K134R; two amines: K121R, K132R, K134R; three amines: K80R, K121R; four amines: K121R; five amines: WT; six amines: N82K, R103K, K121R.

(B) Tetramer integrity of amine series, analyzed by SDS-PAGE with or without boiling and then Coomassie blue staining.

(C) Absorption spectra of amine series after labeling with 635P-NHS, normalized by the 637 nm peak.

(D) Fluorescent brightness of amine series conjugated with 635P-NHS (mean of triplicate ± 1 SD).

(E) Absorption spectra of amine series after labeling with sulfoCy3-NHS, normalized by the 553 nm peak.

(F) Fluorescent brightness of amine series conjugated with sulfoCy3-NHS (mean of triplicate ± 1 SD). See also Figure S3.

(note that we determined the on rate for unlabeled WT streptavidin to be 5.7 ± 0.3 × 10^7 M^{-1} s^{-1}). This dye-labeling effect on the on rate is consistent with the small change upon labeling the K121R streptavidin mutant. Importantly, 635P labeling of Flavidin did not accelerate biotin-conjugate off rate (Figure 4C). After 10 hr, there was 15% ± 0.6% dissociation from Flavidin and 13% ± 2% dissociation from Flavidin-635P. Labeling Flavidin with alternative dyes Atto647N-NHS or Atto590-NHS similarly did not accelerate biotin-conjugate dissociation (Figure S4B). Overall, Flavidin showed high binding stability and thermostability, similar to WT streptavidin, while minimizing the effects of dye labeling on ligand binding.

We also used electrospray ionization mass spectrometry (MS) to analyze labeling by dye. After sulfo-Cy3-NHS labeling, we found a dominant peak at 14,757.2, consistent with the expected single modification per Flavidin monomer (predicted mass 14,756.9) (Figure S5A). There was also a smaller proportion of the Flavidin unlabeled or dual labeled, likely to relate to a low efficiency reaction of NHS-conjugates with non-amine side chains (Figure S5A) (Madler et al., 2009). Further MS analysis showed an expected increase in labeling extent along with amine number, when comparing the one to six amine streptavidin series; WT streptavidin had principally three or four dyes attached per monomer (Figure S5B). Similarly MS revealed the increase in dye stoichiometry as dye:monomer ratio was increased for WT streptavidin (Figure S5C), following the labeling variation explored in Figure 1C.

Flavidin Enhanced Brightness and Staining in Cellular Contexts

Having modified several surface residues of streptavidin, it was important to validate that Flavidin still allowed labeling with good specificity in a cellular context. HeLa cells were stained with a biotinylated affibody specific to the epidermal growth factor receptor (Friedman et al., 2007). The affibody was then detected using flow cytometry with 635P-labeled WT streptavidin or Flavidin. Comparable low background staining was seen with WT-635P (geometric mean fluorescence 0.80) or Flavidin-635P (geometric mean 0.76) (Figure 5A), indicating that Flavidin had no effect on non-specific binding. To understand how Flavidin would perform on cells bearing low levels of biotinylated target, we incubated HeLa cells with a range of affibody concentrations. At the lowest affibody concentration of 3 nM, the signal with WT-635P substantially overlapped with the background signal (geometric mean 1.7), whereas for Flavidin-635P nearly all cells were clearly resolved from the background signal (geometric mean 5.1) (Figure 5A). A substantially enhanced signal with Flavidin compared with WT was seen at all affibody concentrations.

Having confirmed that the brightness enhancement with these dyes was retained in a cellular context, we further evaluated the utility of Flavidin for immunohistochemistry of primary tissue samples. Consecutive tissue sections from mouse spleen were fixed with formaldehyde, permeabilized, and blocked. Next, the sections were incubated with a biotinylated anti-CD3 antibody

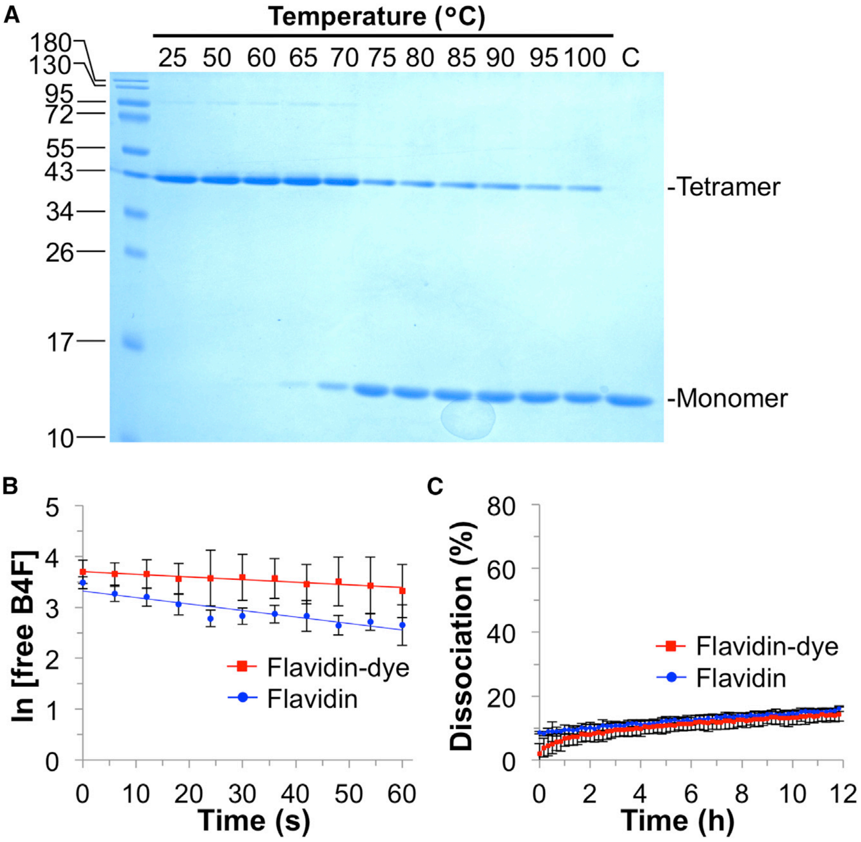

Figure 4. Validation of Flavidin Thermostability and Ligand Binding

(A) Flavidin thermostability after heating for 3 min in PBS at the indicated temperature and then analysis of tetramer integrity by SDS-PAGE with Coomassie blue staining. C is a control boiled in SDS before loading.

(B) Biotin-4-fluorescein association rates for Flavidin with or without 635P-NHS labeling (mean ± 1 SD, n = 9).

(C) Biotin-4-fluorescein dissociation rates for Flavidin with or without 635P-NHS labeling (mean of triplicate ± 1 SD). See also Figure S4.

(for labeling T cell populations), as well as an Alexa Fluor 488-conjugated anti-CD45R/B220 antibody (for B cell populations), followed by staining with either Flavidin or WT streptavidin labeled with either 635P or sulfoCy3. In the case of 635P-labeled samples, the Flavidin panel showed substantially brighter staining of T cells (Figure 5B). Interestingly, sulfoCy3-labeled samples, both WT and Flavidin, showed intense T cell staining (Figure 5C). However, the WT-sulfoCy3 showed undesired non-specific binding, as shown by staining of B cells and diffuse T cell staining. Flavidin-sulfoCy3 gave well-defined and specific staining of T cells (Figure 5C). Thus, in addition to the brightness benefit, Flavidin improved the overall quality of cell staining.

DISCUSSION

Protein modification using NHS probes is a ubiquitous approach, but can have important consequences for both protein and probe function. Dye-NHS labeling of streptavidin slowed ligand association and accelerated dissociation. K121R mutation allowed dye-NHS labeling without any impact on biotin-conjugate dissociation. Landscaping of other amines in streptavidin had a major effect on fluorescent brightness. For 635P and sulfoCy3, we found the highest brightness with the Flavidin mutant, where all lysines were removed. Dye-labeled Flavidin gave strong ligand binding and thermostability, combined with optimal fluorescent brightness and cell staining.

All lysines were previously removed from proteins for site-specific PEGylation (Yamamoto et al., 2003) or to block ubiquitination (Arnason and Ellison, 1994). However, in cases such as GFP, lysines cannot be removed without losing function (Sokalingam et al., 2012). Streptavidin is a highly optimized system and it is easy for mutations to damage assembly or binding (Laitinen et al., 2006). For example, supercharging streptavidin to +52 conferred impressive thermal resilience but weakened ligand binding (Lawrence et al., 2007). Lysines in streptavidin have been modified previously: reaction with succinic anhydride altered accumulation in different organs (Wilbur et al., 2002), while K121 was mutated in converting streptavidin into a catalyst (Jeschek et al., 2016; Pazy et al., 2003).

Ligand-binding affinity is often measured in the best-case scenario, but then generalized to other conditions or ligand-conjugates without necessary caution. Streptavidin:biotin interaction is substantially weakened by endosomal pH (Bruneau et al., 2005), force (Morris et al., 2001; Wong et al., 1999), nanoparticle attachment (by 6 orders of magnitude) (Swift et al., 2006), and bulky biotin-conjugates (Wilbur et al., 2000) (systematically analyzed with Love-Hate ligands) (Fairhead et al., 2014b). Dye labeling must be added to this list, although Flavidin shows how to address this challenge. Such undesired partings also motivated further stabilization of biotin binding, including locking streptavidin's L3/4 loop (Chivers et al., 2010) or mutating R114L in avidin (Taskinen et al., 2014b).

Dye attachment can sterically disrupt protein-ligand interactions, but can also promote new non-specific interactions. Increased non-specific binding correlates with dye hydrophobicity (Pauli et al., 2013) and can alter cellular staining (Hayashi-Takanaka et al., 2014), single-particle mobility (Zanetti-Domingues et al., 2013), and membrane insertion (Hughes et al., 2014). Our histochemistry illustrates how Flavidin improved sensitivity using 635P and specificity using sulfoCy3. Reduced non-specific binding is consistent with the fewer sulfoCy3 molecules attached to Flavidin versus WT streptavidin, as seen by MS. Our amine-landscaping approach makes clear the benefit of minimalist labeling for maximizing specific signal.

Photophysical interactions which may contribute to quenching in dye-labeled WT streptavidin include homo-fluorescence

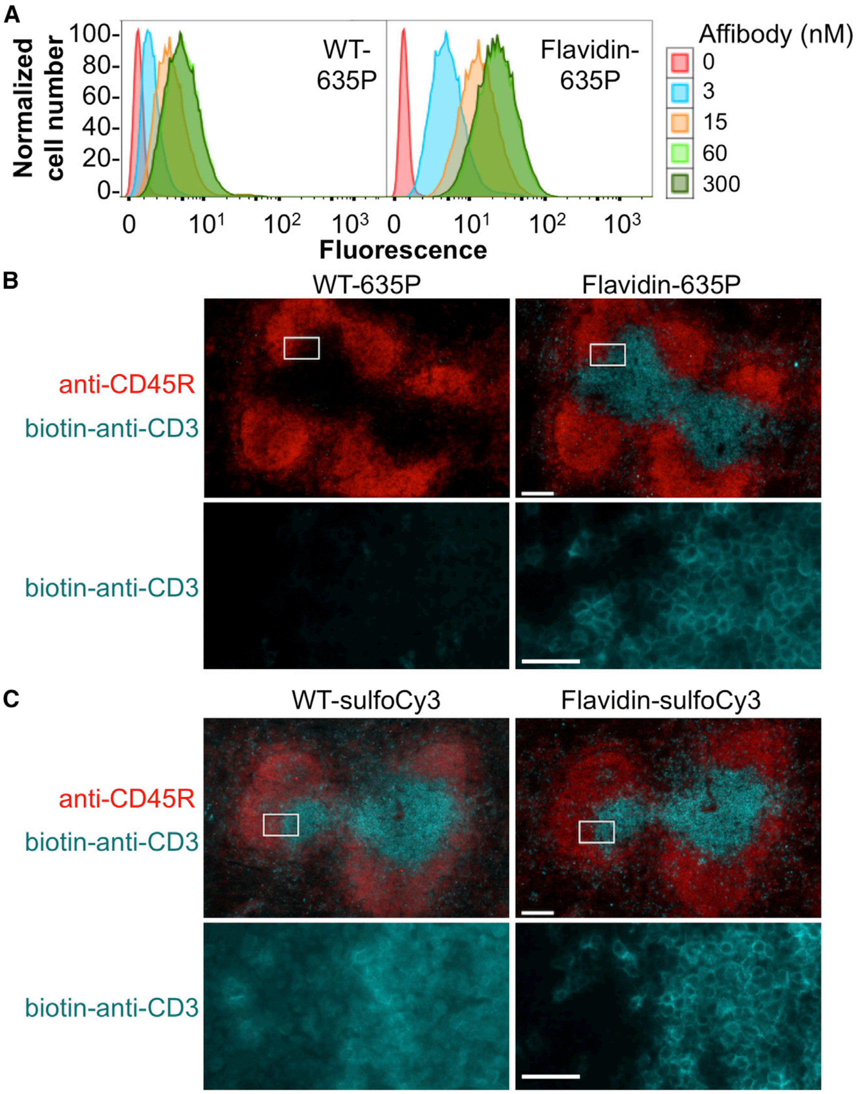

Figure 5. Flavidin Enhanced Specific Cellular Staining

(A) HeLa cells were incubated with varying concentrations of biotinylated affibody against epidermal growth factor receptor and then labeled with WT-635P (left panel) or Flavidin-635P (right panel), before analysis by flow cytometry.

(B) Spleen sections were stained with Alexa Fluor 488-conjugated anti-CD45R antibody (shown in red) and biotinylated anti-CD3 antibody followed by WT-635P or Flavidin-635P (shown in cyan). The bottom row is the inset from the top row, showing only the 635P signal.

(C) As for (B) but with WT-sulfoCy3 or Flavidin-sulfoCy3 (in cyan). Scale bars, 100 μm (top row) or 20 μm (bottom row). See also Figure S5.

resonance energy transfer (homo-FRET, radiationless transfer up to 10 nm dependent on overlap of excitation and emission spectra), photoinduced electron transfer, and exciton coupling (where dyes physically contact) (Doose et al., 2009). Detailed analysis of photophysics for an obligate protein tetramer is beyond the scope here. Also, the mobility of streptavidin's N-terminal amine in crystal structures (Freitag et al., 1999) complicates assignment of specific dye-dye or dye-amino acid distances that could affect brightness. Such interactions may be more efficiently studied on DNA with only two dye attachment sites (Cordes et al., 2010). Nevertheless, the changes in absorption spectrum with increasing amine number point toward physical dye-dye contact contributing to quenching for streptavidin (Pauli et al., 2011; Schobel et al., 2000). Given the relative protein dimensions, the low dye number for optimal streptavidin brightness is consistent with the four to six dyes optimal for antibody brightness (Vira et al., 2010). Brightness is a precious commodity for temporal and spatial resolution in super-resolution microscopy and diagnostic sensitivity (Liu et al., 2015; Martelli et al., 2016). Notably, dye-NHS labeling of streptavidin led to poor brightness and utility in MHC-tetramer monitoring of immune responses (Davis et al., 2011; Ramachandiran et al., 2007). Flavidin may also be advantageous for modification with probes such as PEG or DNA, as well as for chemical conjugation to antibodies or enzymes via NHS-maleimide (Hylarides et al., 2001; Rabe and Niemeyer, 2011; Yamamoto et al., 2003). Amine landscaping may have broad relevance to enhance activity following conjugation, including for widely used platforms such as antibodies and non-immunoglobulin scaffolds (Skrlec et al., 2015).

SIGNIFICANCE

Proteins gain diverse new functions after chemical modification. Modification is most easily achieved through reactive probes coupling to abundant amines on the protein surface. We focus on coupling dyes to streptavidin, a powerful tool because of streptavidin's extraordinary affinity for biotin. We found that dye coupling to streptavidin accelerated ligand dissociation, decreased ligand association, and reduced specificity in cellular environments. By choosing appropriate amine groups to retain on streptavidin, "amine landscaping," we generated a Fluorophore-friendly streptavidin (Flavidin). Flavidin greatly enhanced brightness for biochemical and cellular applications, while retaining the ultrastable binding of unlabeled protein. In addition to upgrading a ubiquitous labeling tool, this work introduces amine landscaping as a strategy to increase fluorescence and functional performance of protein conjugates.

STAR★METHODS

Detailed methods are provided in the online version of this paper and include the following:

- KEY RESOURCES TABLE
- CONTACT FOR REAGENT AND RESOURCE SHARING

- EXPERIMENTAL MODEL AND SUBJECT DETAILS
- METHOD DETAILS
  - Cloning
  - Protein Expression
  - SDS-PAGE and Thermostability Testing
  - Protein-Dye Conjugation
  - Absorbance and Fluorescence Brightness Measurements
  - Off-Rate Measurements
  - On-Rate Measurements
  - Cell Culture and Flow Cytometry
  - Immunofluorescence
  - Sequence Alignments
  - Mass Spectrometry
  - Data and Software Availability
- QUANTIFICATION AND STATISTICAL ANALYSIS
  - Statistical Analysis

SUPPLEMENTAL INFORMATION

Supplemental Information includes five figures and one table and can be found with this article online at http://dx.doi.org/10.1016/j.chembiol.2017.06.015.

AUTHOR CONTRIBUTIONS

M.T.J. performed all experiments, except P.F. carried out immunohistochemical staining and imaging. M.F. identified the effect of dye conjugation on WT streptavidin off rate. M.T.J. and M.H. designed the experiments. M.T.J. and M.H. wrote the paper. All authors analyzed the data.

ACKNOWLEDGMENTS

Funding was provided by the Biotechnology and Biological Sciences Research Council (BBSRC, grants BB/I006303/1 and BB/M02122X/1) (M.T.J., M.F., and M.H.). We thank Chris Willberg (University of Oxford) for assistance with flow cytometry, Oktawia Borkowska and Rod Chalk (University of Oxford) for assistance with mass spectrometry, and Can Buldun (University of Oxford) for providing the biotinylated affibody. M.T.J. and M.H. are inventors on a patent application regarding streptavidin mutants affecting dye labeling, including Flavidin (UK Patent Application No. 1616051.7).

Received: March 16, 2017
Revised: May 15, 2017
Accepted: June 27, 2017
Published: July 27, 2017

REFERENCES

Agarwal, P., and Bertozzi, C.R. (2015). Site-specific antibody-drug conjugates: the nexus of bioorthogonal chemistry, protein engineering, and drug development. Bioconjug. Chem. *26*, 176–192.

Arnason, T., and Ellison, M.J. (1994). Stress resistance in *Saccharomyces cerevisiae* is strongly correlated with assembly of a novel type of multiubiquitin chain. Mol. Cell Biol. *14*, 7876–7883.

Bragg, P.D., and Hou, C. (1975). Subunit composition, function, and spatial arrangement in the Ca2+-and Mg2+-activated adenosine triphosphatases of *Escherichia coli* and *Salmonella typhimurium*. Arch. Biochem. Biophys. *167*, 311–321.

Bruneau, E., Sutter, D., Hume, R.I., and Akaaboune, M. (2005). Identification of nicotinic acetylcholine receptor recycling and its role in maintaining receptor density at the neuromuscular junction in vivo. J. Neurosci. *25*, 9949–9959.

Chilkoti, A., Schwartz, B.L., Smith, R.D., Long, C.J., and Stayton, P.S. (1995a). Engineered chimeric streptavidin tetramers as novel tools for bioseparations and drug delivery. Biotechnology (NY) *13*, 1198–1204.

Chilkoti, A., Tan, P.H., and Stayton, P.S. (1995b). Site-directed mutagenesis studies of the high-affinity streptavidin-biotin complex: contributions of tryptophan residues 79, 108, and 120. Proc. Natl. Acad. Sci. USA *92*, 1754–1758.

Chivers, C.E., Crozat, E., Chu, C., Moy, V.T., Sherratt, D.J., and Howarth, M. (2010). A streptavidin variant with slower biotin dissociation and increased mechanostability. Nat. Methods *7*, 391–393.

Cordes, T., Santoso, Y., Tomescu, A.I., Gryte, K., Hwang, L.C., Camara, B., Wigneshweraraj, S., and Kapanidis, A.N. (2010). Sensing DNA opening in transcription using quenchable Forster resonance energy transfer. Biochemistry *49*, 9171–9180.

Cordes, T., Maiser, A., Steinhauer, C., Schermelleh, L., and Tinnefeld, P. (2011). Mechanisms and advancement of antifading agents for fluorescence microscopy and single-molecule spectroscopy. Phys. Chem. Chem. Phys. *13*, 6699–6709.

Davis, M.M., Altman, J.D., and Newell, E.W. (2011). Interrogating the repertoire: broadening the scope of peptide-MHC multimer analysis. Nat. Rev. Immunol. *11*, 551–558.

Doose, S., Neuweiler, H., and Sauer, M. (2009). Fluorescence quenching by photoinduced electron transfer: a reporter for conformational dynamics of macromolecules. ChemPhysChem *10*, 1389–1398.

Fairhead, M., Krndija, D., Lowe, E.D., and Howarth, M. (2014a). Plug-and-play pairing via defined divalent streptavidins. J. Mol. Biol. *426*, 199–214.

Fairhead, M., Shen, D., Chan, L.K., Lowe, E.D., Donohoe, T.J., and Howarth, M. (2014b). Love-Hate ligands for high resolution analysis of strain in ultra-stable protein/small molecule interaction. Bioorg. Med. Chem. *22*, 5476–5486.

Freitag, S., Le Trong, I., Klumb, L.A., Chu, V., Chilkoti, A., Stayton, P.S., and Stenkamp, R.E. (1999). X-ray crystallographic studies of streptavidin mutants binding to biotin. Biomol. Eng. *16*, 13–19.

Friedman, M., Nordberg, E., Hoiden-Guthenberg, I., Brismar, H., Adams, G.P., Nilsson, F.Y., Carlsson, J., and Stahl, S. (2007). Phage display selection of affibody molecules with specific binding to the extracellular domain of the epidermal growth factor receptor. Protein Eng. Des. Sel. *20*, 189–199.

Hayashi-Takanaka, Y., Stasevich, T.J., Kurumizaka, H., Nozaki, N., and Kimura, H. (2014). Evaluation of chemical fluorescent dyes as a protein conjugation partner for live cell imaging. PLoS One *9*, e106271.

Houk, K.N., Leach, A.G., Kim, S.P., and Zhang, X. (2003). Binding affinities of host-guest, protein-ligand, and protein-transition-state complexes. Angew. Chem. Int. Ed. *42*, 4872–4897.

Hughes, L.D., Rawle, R.J., and Boxer, S.G. (2014). Choose your label wisely: water-soluble fluorophores often interact with lipid bilayers. PLoS One *9*, e87649.

Hylarides, M.D., Mallett, R.W., and Meyer, D.L. (2001). A robust method for the preparation and purification of antibody/streptavidin conjugates. Bioconjug. Chem. *12*, 421–427.

Hytonen, V.P., Maatta, J.A., Kidron, H., Halling, K.K., Horha, J., Kulomaa, T., Nyholm, T.K., Johnson, M.S., Salminen, T.A., Kulomaa, M.S., et al. (2005). Avidin related protein 2 shows unique structural and functional features among the avidin protein family. BMC Biotechnol. *5*, 28.

Jeschek, M., Reuter, R., Heinisch, T., Trindler, C., Klehr, J., Panke, S., and Ward, T.R. (2016). Directed evolution of artificial metalloenzymes for in vivo metathesis. Nature *537*, 661–665.

Kada, G., Falk, H., and Gruber, H.J. (1999). Accurate measurement of avidin and streptavidin in crude biofluids with a new, optimized biotin-fluorescein conjugate. Biochim. Biophys. Acta *1427*, 33–43.

Kuntz, I.D., Chen, K., Sharp, K.A., and Kollman, P.A. (1999). The maximal affinity of ligands. Proc. Natl. Acad. Sci. USA *96*, 9997–10002.

Laitinen, O.H., Hytonen, V.P., Nordlund, H.R., and Kulomaa, M.S. (2006). Genetically engineered avidins and streptavidins. Cell Mol. Life Sci. *63*, 2992–3017.

Lawrence, M.S., Phillips, K.J., and Liu, D.R. (2007). Supercharging proteins can impart unusual resilience. J. Am. Chem. Soc. *129*, 10110–10112.

Liu, Z., Lavis, L.D., and Betzig, E. (2015). Imaging live-cell dynamics and structure at the single-molecule level. Mol. Cell *58*, 644–659.

Maatta, J.A., Helppolainen, S.H., Hytonen, V.P., Johnson, M.S., Kulomaa, M.S., Airenne, T.T., and Nordlund, H.R. (2009). Structural and functional characteristics of xenavidin, the first frog avidin from *Xenopus tropicalis*. BMC Struct. Biol. *9*, 63.

Madler, S., Bich, C., Touboul, D., and Zenobi, R. (2009). Chemical cross-linking with NHS esters: a systematic study on amino acid reactivities. J. Mass Spectrom. *44*, 694–706.

Martelli, C., Lo Dico, A., Diceglie, C., Lucignani, G., and Ottobrini, L. (2016). Optical imaging probes in oncology. Oncotarget *7*, 48753–48787.

Marttila, A.T., Laitinen, O.H., Airenne, K.J., Kulik, T., Bayer, E.A., Wilchek, M., and Kulomaa, M.S. (2000). Recombinant NeutraLite avidin: a non-glycosylated, acidic mutant of chicken avidin that exhibits high affinity for biotin and low non-specific binding properties. FEBS Lett. *467*, 31–36.

Morris, P.D., Tackett, A.J., and Raney, K.D. (2001). Biotin-streptavidin-labeled oligonucleotides as probes of helicase mechanisms. Methods *23*, 149–159.

Nordlund, H.R., Hytonen, V.P., Laitinen, O.H., and Kulomaa, M.S. (2005). Novel avidin-like protein from a root nodule symbiotic bacterium, *Bradyrhizobium japonicum*. J. Biol. Chem. *280*, 13250–13255.

Papadopoulos, J.S., and Agarwala, R. (2007). COBALT: constraint-based alignment tool for multiple protein sequences. Bioinformatics *23*, 1073–1079.

Pauli, J., Grabolle, M., Brehm, R., Spieles, M., Hamann, F.M., Wenzel, M., Hilger, I., and Resch-Genger, U. (2011). Suitable labels for molecular imaging – influence of dye structure and hydrophilicity on the spectroscopic properties of IgG conjugates. Bioconjug. Chem. *22*, 1298–1308.

Pauli, J., Licha, K., Berkemeyer, J., Grabolle, M., Spieles, M., Wegner, N., Welker, P., and Resch-Genger, U. (2013). New fluorescent labels with tunable hydrophilicity for the rational design of bright optical probes for molecular imaging. Bioconjug. Chem. *24*, 1174–1185.

Pazy, Y., Raboy, B., Matto, M., Bayer, E.A., Wilchek, M., and Livnah, O. (2003). Structure-based rational design of streptavidin mutants with pseudo-catalytic activity. J. Biol. Chem. *278*, 7131–7134.

Rabe, K.S., and Niemeyer, C.M. (2011). Selective covalent conjugation of phosphorothioate DNA oligonucleotides with streptavidin. Molecules *16*, 6916–6926.

Ramachandiran, V., Grigoriev, V., Lan, L., Ravkov, E., Mertens, S.A., and Altman, J.D. (2007). A robust method for production of MHC tetramers with small molecule fluorophores. J. Immunol. Methods *319*, 13–20.

Sano, T., and Cantor, C.R. (1995). Intersubunit contacts made by tryptophan 120 with biotin are essential for both strong biotin binding and biotin-induced tighter subunit association of streptavidin. Proc. Natl. Acad. Sci. USA *92*, 3180–3184.

Sano, T., Pandori, M.W., Chen, X., Smith, C.L., and Cantor, C.R. (1995). Recombinant core streptavidins. A minimum-sized core streptavidin has enhanced structural stability and higher accessibility to biotinylated macromolecules. J. Biol. Chem. *270*, 28204–28209.

Sano, T., Vajda, S., Reznik, G.O., Smith, C.L., and Cantor, C.R. (1996). Molecular engineering of streptavidin. Ann. N. Y. Acad. Sci. *799*, 383–390.

Sano, T., Vajda, S., and Cantor, C.R. (1998). Genetic engineering of streptavidin, a versatile affinity tag. J. Chromatogr. B Biomed. Sci. Appl. *715*, 85–91.

Schobel, U., Egelhaaf, H.J., Frohlich, D., Brecht, A., Oelkrug, D., and Gauglitz, G. (2000). Mechanisms of fluorescence quenching in donor-acceptor labeled antibody-antigen conjugates. J. Fluoresc *10*, 147–154.

Shinmi, D., Taguchi, E., Iwano, J., Yamaguchi, T., Masuda, K., Enokizono, J., and Shiraishi, Y. (2016). One-step conjugation method for site-specific antibody-drug conjugates through reactive cysteine-engineered antibodies. Bioconjug. Chem. *27*, 1324–1331.

Skrlec, K., Strukelj, B., and Berlec, A. (2015). Non-immunoglobulin scaffolds: a focus on their targets. Trends Biotechnol. *33*, 408–418.

Sokalingam, S., Raghunathan, G., Soundrarajan, N., and Lee, S.G. (2012). A study on the effect of surface lysine to arginine mutagenesis on protein stability and structure using green fluorescent protein. PLoS One *7*, e40410.

Stephanopoulos, N., and Francis, M.B. (2011). Choosing an effective protein bioconjugation strategy. Nat. Chem. Biol. *7*, 876–884.

Swift, J.L., Heuff, R., and Cramb, D.T. (2006). A two-photon excitation fluorescence cross-correlation assay for a model ligand-receptor binding system using quantum dots. Biophys. J. *90*, 1396–1410.

Takakura, Y., Oka, N., Kajiwara, H., Tsunashima, M., Usami, S., Tsukamoto, H., Ishida, Y., and Yamamoto, T. (2010). Tamavidin, a versatile affinity tag for protein purification and immobilization. J. Biotechnol. *145*, 317–322.

Taskinen, B., Airenne, T.T., Janis, J., Rahikainen, R., Johnson, M.S., Kulomaa, M.S., and Hytonen, V.P. (2014a). A novel chimeric avidin with increased thermal stability using DNA shuffling. PLoS One *9*, e92058.

Taskinen, B., Zauner, D., Lehtonen, S.I., Koskinen, M., Thomson, C., Kahkonen, N., Kukkurainen, S., Maatta, J.A., Ihalainen, T.O., Kulomaa, M.S., et al. (2014b). Switchavidin: reversible biotin-avidin-biotin bridges with high affinity and specificity. Bioconjug. Chem. *25*, 2233–2243.

Veggiani, G., Nakamura, T., Brenner, M.D., Gayet, R.V., Yan, J., Robinson, C.V., and Howarth, M. (2016). Programmable polyproteams built using twin peptide superglues. Proc. Natl. Acad. Sci. USA *113*, 1202–1207.

Vira, S., Mekhedov, E., Humphrey, G., and Blank, P.S. (2010). Fluorescent-labeled antibodies: balancing functionality and degree of labeling. Anal. Biochem. *402*, 146–150.

Wang, L., Amphlett, G., Blattler, W.A., Lambert, J.M., and Zhang, W. (2005). Structural characterization of the maytansinoid-monoclonal antibody immunoconjugate, huN901-DM1, by mass spectrometry. Protein Sci. *14*, 2436–2446.

Wilbur, D.S., Chyan, M.K., Pathare, P.M., Hamlin, D.K., Frownfelter, M.B., and Kegley, B.B. (2000). Biotin reagents for antibody pretargeting. 4. Selection of biotin conjugates for in vivo application based on their dissociation rate from avidin and streptavidin. Bioconjug. Chem. *11*, 569–583.

Wilbur, D.S., Hamlin, D.K., Meyer, D.L., Mallett, R.W., Quinn, J., Vessella, R.L., and Press, O.W. (2002). Streptavidin in antibody pretargeting. 3. Comparison of biotin binding and tissue localization of 1,2-cyclohexanedione and succinic anhydride modified recombinant streptavidin. Bioconjug. Chem. *13*, 611–620.

Wong, J., Chilkoti, A., and Moy, V.T. (1999). Direct force measurements of the streptavidin-biotin interaction. Biomol. Eng. *16*, 45–55.

Wu, Y., Wu, X., Lu, R., Zhang, J., Toro, L., and Stefani, E. (2015). Resonant scanning with large field of view reduces photobleaching and enhances fluorescence yield in STED microscopy. Sci. Rep. *5*, 14766.

Yamamoto, Y., Tsutsumi, Y., Yoshioka, Y., Nishibata, T., Kobayashi, K., Okamoto, T., Mukai, Y., Shimizu, T., Nakagawa, S., Nagata, S., et al. (2003). Site-specific PEGylation of a lysine-deficient TNF-alpha with full bioactivity. Nat. Biotechnol. *21*, 546–552.

Zanetti-Domingues, L.C., Tynan, C.J., Rolfe, D.J., Clarke, D.T., and Martin-Fernandez, M. (2013). Hydrophobic fluorescent probes introduce artifacts into single molecule tracking experiments due to non-specific binding. PLoS One *8*, e74200.

STAR★METHODS

KEY RESOURCES TABLE

REAGENT or RESOURCE	SOURCE	IDENTIFIER
Antibodies		
biotinylated anti-CD3 antibody	BioLegend	clone 17A2
AlexaFluor 488-conjugated anti-CD45R/ B220 antibody	BioLegend	clone RA3-6B2
Bacterial and Virus Strains		
E. coli DH5α cells	Thermo Scientific	18265017
E. coli BL21 DE3 RIPL cells	Agilent	230280
Biological Samples		
Spleen from a 12 week-old wild-type C57BL/6 mouse	Charles River	Strain 027
Chemicals, Peptides, and Recombinant Proteins		
Biotin	Sigma-Aldrich	B4501
biotin-4-fluorescein	Sigma-Aldrich	B9431
Abberior STAR 635P NHS carbonate	Abberior	1-0101-007-6C
sulfo-Cyanine 3 (sulfoCy3) NHS ester	Abcam	ab146458
Atto647N NHS ester	Atto-Tec	AD 647N-31
Atto590 NHS ester	Atto-Tec	AD 590-31
avidin/biotin blocking kit	Vector laboratories	SP-2001
4',6-diamidino-2-phenylindole (DAPI)	Sigma-Aldrich	D9542
ProLong Gold mounting media	Thermo Fisher Scientific	P36930
Critical Commercial Assays		
Pierce Microplate bicinchoninic acid (BCA) Protein Assay Kit	Thermo Fisher Scientific	23235
Deposited Data		
Nucleotide sequence of Flavidin	This paper	GenBank accession number MF150043
Nucleotide sequence of K121R Streptavidin	This paper	GenBank accession number MF150042
Experimental Models: Cell Lines		
HeLa (ATCC® CCL-2™) cell line	ATCC	ATCC® CCL-2™
Oligonucleotides		
See Table S1 for oligonucleotide sequences	-	-
Recombinant DNA		
pET21_Streptavidin-E6	Fairhead et al. (2014a)	Addgene Plasmid: #46367
pET21_Streptavidin-K121R	This paper	Addgene Plasmid: #89880
pET21_Flavidin	This paper	Addgene Plasmid: #89881
Software and Algorithms		
COBALT Constraint-Based Multiple Protein Alignment Tool	Papadopoulos and Agarwala (2007)	https://www.ncbi.nlm.nih.gov/tools/cobalt/re_cobalt.cgi
FlowJo v10	Tree Star Inc	https://www.flowjo.com/
ChemDraw Professional 16.0	PerkinElmer	http://www.cambridgesoft.com/Ensemble_for_Chemistry/ChemDraw/ChemDrawProfessional/
Image Lab v5.2.1	Bio-Rad	http://www.bio-rad.com/en-uk/product/image-lab-software
VSViewer	MetaSystems	https://metasystems-international.com/en/products/metafer/
MassHunter Qualitative Analysis	Agilent	http://www.agilent.com//en-us/products/software-informatics/masshunter-suite

CONTACT FOR REAGENT AND RESOURCE SHARING

Further information and request for resources and reagents should be directed to and will be fulfilled by the Lead Contact, Mark Howarth (mark.howarth@bioch.ox.ac.uk).

Requests for plasmids for Flavidin (pET21-Flavidin, https://www.addgene.org/89881/) and K121R mutant (pET21-Streptavidin-K121R, https://www.addgene.org/89880/) may also be made from Addgene. These materials will be released subject to the Uniform Biological Material Transfer Agreement of Addgene.

EXPERIMENTAL MODEL AND SUBJECT DETAILS

See Method Details below for details on cell cultures and culture conditions

METHOD DETAILS

Cloning

PCR was performed using KOD Hot Start DNA Polymerase (Merck Chemicals). Gibson Assembly Master Mix (New England BioLabs) was used according to the manufacturer's instructions. Constructs were initially cloned into chemically competent *E. coli* DH5α cells. All mutants were verified by sequencing of the entire gene. pET21 core streptavidin encoding a C-terminal hexaglutamate tag (SAe, "WT" or "5-amine") (GenBank accession number KF378616, Addgene plasmid #46367) was used as the starting template for new streptavidins.

pET21 SAe-K121R ("K121R" or "4-amine") (GenBank accession number MF150042, Addgene plasmid #89880) was generated by QuikChange site-directed mutagenesis on SAe using primer 5'-GCTAACGCGTGGCGATCCACCCTGGTTGG and its reverse complement.

pET21 SAe-K121Q was generated by QuikChange on SAe using primer 5'-GCTAACGCGTGGCAATCCACCCTGGTTGG and its reverse complement.

pET21 SAe-K121E was generated by QuikChange on SAe using primer 5'-GCTAACGCGTGGGAATCCACCCTGGTTGG and its reverse complement.

pET21 SAe-K121A was generated by QuikChange on SAe using primer 5'-GCTAACGCGTGGGCATCCACCCTGGTTGG and its reverse complement.

pET21 SAe-K121R-N82K-R103K ("6-amine") was generated in two steps. First N82K was introduced into SAe-K121R by Gibson assembly of two PCR products: (product 1) PCR with primers 5'- CCGTTGCTTGGAAAAACAAATACCGTAACGCTCACTCCGCTAC CACC and 5'- GATCGTTGTCAGAAGTAAGTTGGCC (AmpB), (product 2) PCR with primers 5'- GTTTTTCCAAGCAACGGTCCAACC and 5'-GGCCAACTTACTTCTGACAACGATC (AmpA). In the second step, R103K was introduced into pET21 SAe-K121R-N82K by Gibson assembly of two PCR products: (product 1) PCR with primers 5'- CAGTACGTTGGTGGTGCTGAAGCTAAAATCAACACC CAGTGGTTGTTGACC and AmpB, (product 2) PCR with primers 5'- ACCACCAACGTACTGGCCAGACC and AmpA.

pET21 SAe-K121R-K80R ("3-amine") was introduced into SAe-K121R by Gibson assembly of two PCR products: (product 1) PCR with primers 5'- TCTGGGTTGGACCGTTGCTTGGCGCAACAACTACCGTAACGCTCAC and AmpB, (product 2) PCR with primers 5'-CCAAGCAACGGTCCAACCCAGAG and AmpA.

pET21 SAe-K121R-K132R-K134R ("2-amine") was introduced into SAe-K121R template by Gibson assembly of two PCR products: (product 1) PCR with 5'- GGTCACGACACCTTCACCCGTGTTCGTCCGTCCGCTGCTTCCG and AmpB, (product 2) PCR with 5'- GGTGAAGGTGTCGTGACCAACC and AmpA.

pET21 SAe-K121R-K80R-K132R-K134R ("1-amine", Flavidin) (GenBank accession number MF150043, Addgene plasmid #89881) was introduced into the SAe-K121R-K132R-K134R template by Gibson assembly using the two reactions described above for generating pET21 SAe-K121R-K80R. WT core streptavidin and Flavidin amino acid sequences are aligned in Figure S4A.

Protein Expression

Overnight cultures of streptavidin variants were grown at 37 °C and 220 rpm in 10 mL LB with 100 μg/mL ampicillin and 0.8% w/v glucose, after picking a freshly-transformed colony of *E. coli* BL21 DE3 RIPL cells (Agilent). The overnight culture was then diluted to 1 L and grown at 37 °C and 200 rpm to OD_{600} 0.8-1.0. Expression was induced with 0.4 mM IPTG. The culture was incubated for another 4 h at 37 °C. The cell pellet was dissolved in 15 mL 100 mM Tris HCl pH 8.0 and frozen at -80 °C. After thawing, 15 μL 100 mg/mL lysozyme was added and the tube was rocked at 25 °C for 45 min. 750 μL 10% v/v Triton X-100 was added and the pellet was again frozen at -80 °C. After thawing in a 25 °C water bath, 15 mL milliQ water was added and the solution was vortexed for 30 s. This solution was thrice sonicated on ice for 30 s. The cell lysate was centrifuged at 20,000 g for 20 min. The pellet was washed thrice with 25 mL milliQ water, dissolved in 10 mL 6 M guanidinium hydrochloride pH 1.5 and centrifuged at 20,000 g for 20 min. The supernatant was then added drop-by-drop to 200 mL fast-stirring PBS (137 mM NaCl, 2.7 mM KCl, 10 mM Na_2HPO_4, 1.8 mM KH_2PO_4 pH 7.4) at 4 °C. After 16 h, folded streptavidin variant was precipitated by slow addition of 120 g ammonium sulfate at 4 °C, followed by incubation for an additional 60 min. The pellet was isolated by centrifugation at 20,000 g for 20 min and dissolved in 10 mL 20 mM Tris HCl pH 8.0. A further centrifugation was performed at 20,000 g for 20 min to remove insoluble protein.

The supernatant, containing the streptavidin variant, was dialyzed overnight into PBS (three buffer exchanges) and centrifuged at 4,700 g for 10 min. Concentrations of protein before dye modification were determined by absorbance at 280 nm and are given as the tetramer concentrations.

SDS-PAGE and Thermostability Testing

SDS-PAGE was performed on 16% polyacrylamide gels using the XCell SureLock system (Thermo Fisher Scientific). Non-boiled samples were mixed with 6× SDS buffer (0.23 M Tris HCl pH 6.8, 24% v/v glycerol, 120 μM bromophenol blue, 0.23 M SDS) and directly loaded, while boiled samples were mixed with 6× SDS buffer, heated at 95 °C for 5 min, and then loaded. Gels were run at 190 V, with the gel box packed in ice to minimize denaturation of folded tetramers. Gels were stained with InstantBlue (Expedeon) and images were collected using a ChemiDoc XRS+ system with Image Lab v5.2.1 software (Bio-Rad).

To analyze tetramer stability, we incubated 4 μM streptavidin variant in PBS for 3 min at the specified temperature, followed by cooling to 10 °C, using the Bio-Rad C1000 Thermal Cycler. Samples were then mixed with 6× SDS buffer and promptly loaded on SDS-PAGE.

Protein-Dye Conjugation

10 μL freshly-prepared 1 M $NaHCO_3$ pH 8.3 was added to 100 μL 10 μM streptavidin variant in PBS. This solution was added to 5 μL 10 mg/mL dye-NHS ester in dry dimethylsulfoxide (DMSO). The tested dyes were Abberior STAR 635P NHS carbonate (Abberior), sulfo-Cyanine 3 NHS ester (Abcam), Atto647N NHS ester (ATTO-TEC), and Atto590 NHS ester (ATTO-TEC). This protocol gave a 635P-NHS:streptavidin monomer ratio of 12:1 or a sulfoCy3-NHS:streptavidin monomer ratio of 17:1. In experiments where the dye:protein ratio was varied, 5.0, 2.5, 1.0 or 0.5 μL of 10 mg/mL dye-NHS was added to the solution of 100 μL 10 μM WT streptavidin in PBS with 10 μL freshly-prepared 1 M $NaHCO_3$ pH 8.3.

The reaction was incubated at 25 °C with end-over-end rotation. After 4 h, the reaction was spun at 16,900 g for 5 min. The supernatant was applied to a PBS-washed 0.8 mL slurry of Sephadex G-25 resin. The first two 0.2 mL fractions were pooled and dialyzed thrice, each for >4 h, in 3,000 molecular weight cut-off tubing into PBS at 4 °C. The labeled protein was then spun at 16,900 g for 5 min to remove aggregates. At all steps, the sample was covered by aluminum foil. Protein concentrations were determined using Pierce Microplate bicinchoninic acid (BCA) Protein Assay Kit (Thermo Scientific) on a SpectraMax M3 plate-reader (Molecular Devices). BCA values were corrected for any contribution of dye absorbance by subtracting A_{562} of BCA–untreated controls.

Absorbance and Fluorescence Brightness Measurements

Fluorescent brightness of labeled streptavidin variants was measured in clear-bottom black-wall 96-well plates on a SpectraMax M3 plate-reader. For 635P-labeled samples, protein concentration of 1 μM, excitation of 620 nm, filter cut-off of 630 nm, and emission of 660 nm were used. Measurement was also repeated with 0.1 μM protein. For sulfo-Cy3-labeled samples, protein concentration of 1 μM, excitation of 545 nm, cut-off of 550 nm, and emission of 570 nm were used. Fluorescent brightness is reported in arbitrary units.

Absorbance spectra were acquired on an ND-1000 Spectrophotometer (Thermo Scientific) using software version 3.8.1, at protein concentrations of 1 μM, with the obtained absorbance values <0.3 to minimize inner-filter effects. Absorbance spectra were normalized by setting the values at the absorption maximum equal to 1. All fluorescence brightness and absorbance spectra readings were performed at 25 °C in PBS.

Off-Rate Measurements

Biotin-4-fluorescein (B4F) off-rate experiments were performed on a TECAN SpectraFluor Plus plate-reader, using 484 nm excitation and 535 nm emission. In clear 96-well plates, we added 10 μL 1 μM streptavidin variant in PBS to 170 μL 24 nM B4F in PBS, followed by incubation at 37 °C for 1 h. Excess biotin (20 μL 1 mM biotin in PBS) was added and fluorescence time-points were immediately acquired at 37 °C. Two controls were performed in parallel. In the quenched control, 10 μL 1 μM streptavidin variant in PBS was added to 170 μL of 24 nM B4F in PBS, followed by addition of 20 μL of PBS. In the "B4F only" control, 10 μL PBS was added to 170 μL 24 nM B4F in PBS, followed by addition of 20 μL 1 mM biotin in PBS. The percent B4F dissociation was calculated using: 100× [(streptavidin with B4F)-(quenched control)]/[(B4F only control)-(quenched control)]. Data are means and standard deviations of three experiments.

On-Rate Measurements

B4F on-rate experiments were performed on a PHERAstar RS plate reader using 485 nm excitation and 520 nm emission. In clear-bottom black-wall 96-well plates, 100 μL 500 pM streptavidin variant in PBS was added to 100 μL 100 pM B4F in PBS. Fluorescence readings at 25 °C were started immediately. Two controls were performed in parallel. For the "streptavidin only" control, 100 μL 500 pM streptavidin variant in PBS was added to 100 μL PBS. In the "B4F only" control, 100 μL of PBS was added to 100 μL 100 pM B4F in PBS. The fraction of free B4F (over 125 s) was calculated using: [(streptavidin with B4F signal)-(streptavidin only signal)]/[(B4F only signal)-(streptavidin only signal)]. The concentration of free B4F was calculated using: starting B4F concentration of 50 pM × (fraction of free B4F). We plotted ln [free B4F] over time (the first 60 s), where the slope of the line is equal to k_{on} × [streptavidin] concentration of 250 pM. Data were fit using the LINEST function in Microsoft Excel and are means and standard deviations of three experiments, with three replicates per experiment.

Cell Culture and Flow Cytometry

HeLa CCL-2 cells were obtained from the American Type Culture Collection (ATCC) and grown at 37 °C with 5% CO_2 in Dulbecco's Modified Eagle Medium (DMEM, Thermo Fisher Scientific) supplemented with 10% v/v Fetal Bovine Serum, 50 U/mL penicillin, and 50 μg/mL streptomycin (Sigma-Aldrich).

Cells were trypsinized with 0.05% w/v Trypsin-EDTA (Thermo Fisher Scientific), washed with FACS buffer (PBS with 1% w/v bovine serum albumin and 0.1% w/v sodium azide) and hereafter maintained at 4 °C. Cells (500,000/well) were incubated for 20 min with a biotinylated affibody against EGFR [biotin-AP-SnoopTag-AffiEGFR-SpyTag, produced as described (Veggiani et al., 2016)] in FACS buffer at 0, 3, 15, 60 or 300 nM. Cells were then washed twice with FACS buffer. Next, cells were treated with 5 nM WT-635P or Flavidin-635P (dye:protein monomer ratio of 12:1) in FACS buffer for 20 min. Cells were then washed thrice with FACS buffer. Flow cytometry was performed on a MACSQuant Analyzer 10 (Miltenyi Biotech), with excitation at 635 nm and emission at 655-730 nm. Data were analyzed using FlowJo v10 (Tree Star Inc.) and the signal was normalized to the mode cell count for each cell sample.

Immunofluorescence

The spleen from a 12 week-old wild-type C57BL/6 mouse was embedded in optimal cutting temperature compound (O.C.T, Tissue Tec®, Sakura Finetek), and frozen in liquid nitrogen. Tissue sections (10 μm) were cut using a CM3050S cryostat (Leica), dried for 30 min at 22 °C, and stored at -20 °C until use. Sections were fixed in PBS with 2% w/v formaldehyde (Sigma-Aldrich) for 4 min at 22 °C and all further steps were performed at this temperature. Cells in the tissue sections were permeabilized using PBS with 0.1% v/v Triton X-100 (Sigma-Aldrich) for 4 min. The tissue sections were then treated with avidin/biotin blocking kit (Vector laboratories, SP-2001) according to the manufacturer's protocol, followed by PBS + 0.5% w/v bovine serum albumin (Sigma-Aldrich) for 15 min. The sections were then incubated with biotinylated anti-CD3 antibody (1:50 dilution, clone 17A2, BioLegend) and AlexaFluor 488-conjugated anti-CD45R/B220 antibody (1:50 dilution, clone RA3-6B2, BioLegend) in PBS + 0.5% w/v bovine serum albumin for 1 h, followed by 635P- or sulfoCy3-labeled Flavidin or WT streptavidin (0.094 μM, dye:protein monomer ratio of 12:1 for 635P and 17:1 for sulfoCy3) in PBS + 0.1% w/v bovine serum albumin for 30 min, with finally 4',6-diamidino-2-phenylindole (DAPI, 2 μg/mL, Sigma-Aldrich) in PBS for 2 min. Between all steps, the sections were washed thrice with PBS. Slides were mounted using ProLong Gold mounting media (Thermo Fisher Scientific). Images were acquired with a MetaSystems automated slide scanner equipped with a Zeiss AxioImager.Z2 epifluorescence microscope using a Plan-Apochromat 40×/1.4 oil objective lens (Carl Zeiss Microscopy). For sulfoCy3, the excitation interval was 546/10 nm, the emission interval was 575/15 nm, and the exposure time was 33.2 ms. For 635P, the excitation interval was 581/10 nm, the emission interval was 640/30 nm, and the exposure time was 400 ms. The images were extracted from MetaSystems image viewer program VSViewer into Adobe Photoshop. All images shown side-by-side were acquired and analyzed under identical conditions.

Sequence Alignments

Sequence alignment of streptavidin-related tetramers was performed using the online COBALT Constraint-Based Multiple Protein Alignment Tool.

Mass Spectrometry

WT streptavidin or Flavidin was labeled with sulfoCy3-NHS as above, at a 17:1 dye:monomer ratio unless indicated. Samples at 1 μM in PBS were dialyzed thrice, each for >4 h, into 50 mM ammonium acetate. Proteins were spun at 16,900 g for 5 min at 4 °C to remove potential aggregates. Samples were then analyzed on a 6530 electrospray ionization quadrupole time-of-flight mass spectrometer (Agilent Technologies) by direct infusion into the source at 6 μL/min. Data were collected in positive mode using drying gas flow of 5 L/min at 325 °C, with fragmentor voltage of 400 V and skimmer voltage of 65 V. Deconvolution and calculation of peak area were performed using Agilent MassHunter Qualitative Analysis software. Predicted mass was obtained for protein using ExPASy ProtParam, with the N-terminal methionine removed, and for dye using ChemDraw Professional 16.0.

Data and Software Availability

The accession number for the K121R Streptavidin sequence reported in this paper is GenBank MF150042. The accession number for the Flavidin sequence reported in this paper is GenBank MF150043.

QUANTIFICATION AND STATISTICAL ANALYSIS

Statistical Analysis

Off-rate measurements and fluorescent brightness values were means and standard deviations of three experiments analyzed using Microsoft Excel. On-rate measurements were means and standard deviations of nine experiments analyzed using Microsoft Excel.

Cell Chemical Biology, Volume *24*

Supplemental Information

Amine Landscaping to Maximize Protein-Dye Fluorescence and Ultrastable Protein-Ligand Interaction

Michael T. Jacobsen, Michael Fairhead, Per Fogelstrand, and Mark Howarth

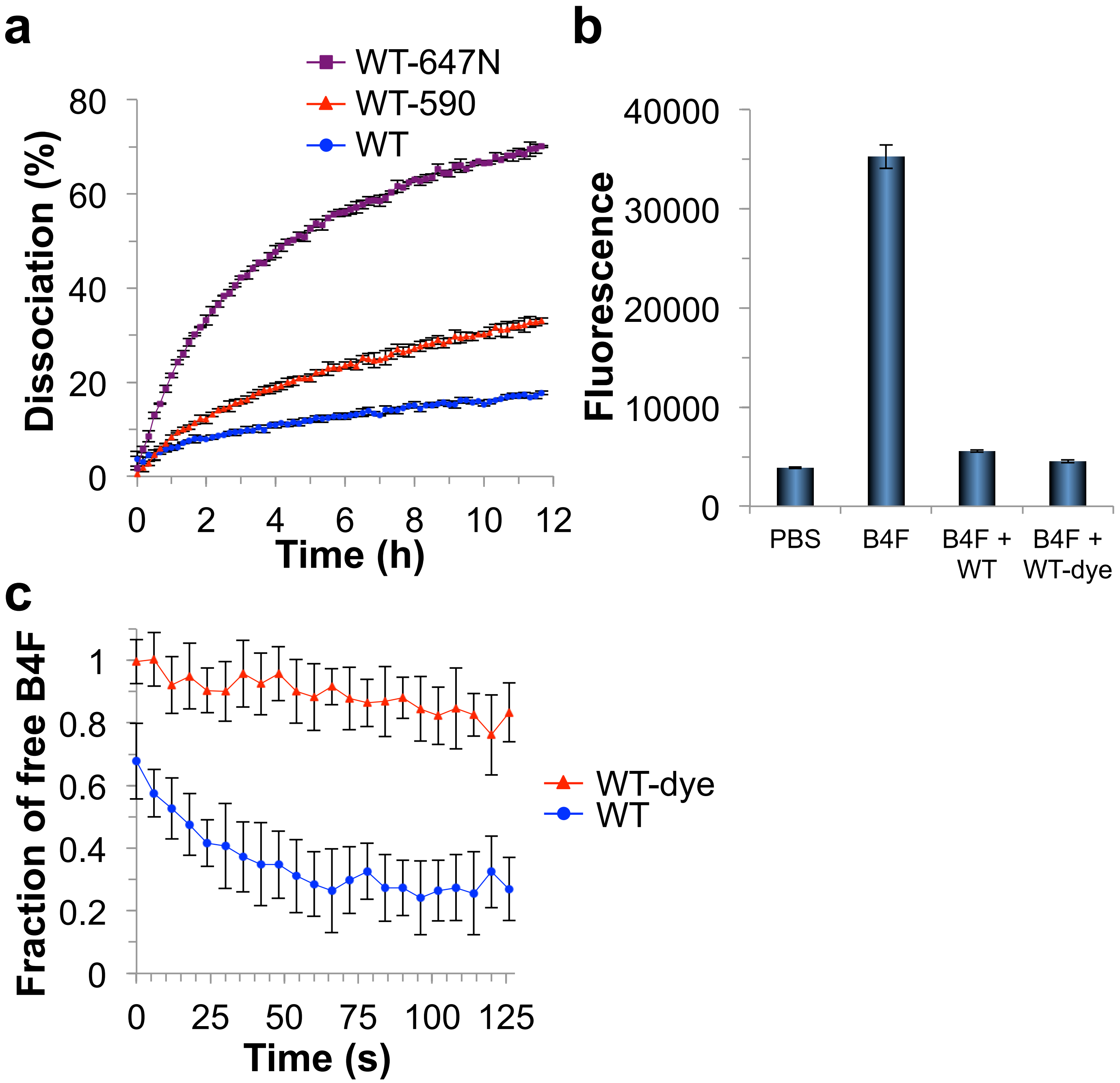

Fig. S1 (related to Fig. 1). Dye labeling of streptavidin impaired ligand binding. (a) Increased dissociation rate after labeling streptavidin with alternative dyes. Biotin-4-fluorescein (B4F) dissociation rates for WT streptavidin unmodified or after conjugation with Atto647N-NHS or Atto590-NHS (mean of triplicate ± 1 s.d.). (b) Streptavidin conjugation with dye had little effect on initial B4F quenching. Raw fluorescence values (arbitrary units) in the B4F dissociation rate assay for WT streptavidin and 635P-NHS-labeled WT streptavidin, immediately before addition of excess biotin, from Fig. 1a (mean ± 1 s.d., n=3). Controls with PBS alone or B4F alone are included. (c) Labeling slowed ligand binding by streptavidin. Fraction of free B4F after incubation with WT streptavidin or 635P-NHS-labeled streptavidinin. Compared to Fig. 1b, the data are shown for a longer time and with a linear y-axis (mean ± 1 s.d., n=9).

a

```
Streptavidin        ---MRKIVVAAIAVSLTTVSITASASADPSKD-----SKAQVSAAEAGITGTWYNQLGSTFIVTAG-A-D
Avidin              -----------MVHATSPLLLLLLLSLALVAPG--------LSARKCSLTGKWTNDLGSNMTIGAVNS-R
Neutralite Avidin   -----------MVHATSPLLLLLLLSLALVAPG--------LSAAICSLTGEWTNDLGSIMTIGAVNS-N
Bradavidin I        ------------MRHFNGMLLAMIASTSLIGP---------LPAYAQSVNWTWTNQYGSTLAITSFNSNT
Tamavidin 1         -----------------------------------------MKDVQSLLTGTWYNELGSTMNLTAN-K-D
Tamavidin 2         -----------------------------------------MSDVQSSLTGTWYNELNSKMELTAN-K-D
Xenavidin           ------------MNALTLLAVALALYGCICTE-----------AQKCNLQGQWRNKLGSNLIIESV-SQN
S. virginiae        ---MRKIVVAAIAVSLTTISITASASADPSKD-----SKAQVSAAEAGITGTWYNQLGSTFIVTAG-A-D
S. katrae           ---MRKIVVAAIAVSLTTVGIAANASADPAKD-----SKAQVAAVDAGITGTWYNQLGSTFTVTAN-T-D
S. flavotricini     MTRVRQTLVALCALSLTFVTVSASASADTRATVSQTAESGSVTESRPGILGTWYNQLGSVMVVTRA-A-N
S. hygroscopicus    ----------------MTVEVTVRASREKIMP----------------IDGEWYNEFGSQVRLESA-P-E

Streptavidin        GALTGTYESAVGNAESRYVLTGRYDSAPATDG-SGTALGWTVAWKNNYRNAHSATTWSGQ--YVGGAEAR
Avidin              GEFTGTYITAVTATSNEIKESPLHGTQNTINKRTQPTFGFTVNWK----FSESTTVFTGQCFIDRNGKEV
Neutralite Avidin   GEFTGTYITAVTATSNEIKESPLHGTQNTINKATQPTFGFTVNWK----FSESTTVFTGQCFIDRNGKEV
Bradavidin I        GAITGTYTNNAANSCDEGKPQGVTGWLAYGNT--GTAISFSVNFL----GCGSTTVWTGQ----LNNATG
Tamavidin 1         GSLTGTYHSNVGEVPPTYHLSGRYNLQPPSGQ--GVTLGWAVSFENTSANVHSVSTWSGQ--YFSEPAEV
Tamavidin 2         GTLTGKYLSKVGDVYVPYPLSGRYNLQPPAGQ--GVALGWAVSWEN--SKIHSATTWSGQ--FFSESSPV
Xenavidin           GEFTGTYFTSVSLTNSTIRISPLTGYQKLTEK---PTFGFTVHWA----FSDSITVWTGQCFLNEKGEEI
S. virginiae        GALTGTYESAVGNAESRYVLTGRYDSTPATDG-SGTALGWSVAWKNNYRNAHSATTWSGQ--YVGGAEAR
S. katrae           GSLTGTYESAVGNAESRYVVTGRYDTAAAT-G-TGTALGWTVAWKNDFRNAHSATTWSGQ--YVGGADAK
S. flavotricini     GGFTGTYESAVGNAEKRYTMTGRYDSAPAD-G-TGTAVGWTVAYRNAHRNAHSVATWSGQ--YVGGSQER
S. hygroscopicus    GTVSGEYVTAVGHAPGTYLLTGRHDG-PTSPG-NGIALGWTVAWRNEHGNLGSVTSWSGL---FLEGPER

Streptavidin        INTQWLLTSGT-TEANAWKSTLVGHDTFTKVKPSAASIDAAKKAGVNNGNPLDAVQQ
Avidin              LKTMWLLRSSVNDIGDDWKATRVGINIFTRLRTQKE---------------------
Neutralite Avidin   LKTMWLLRSSVNDIGDDWKATRVGINIFTALATQKE---------------------
Bradavidin I        FQGLWYLSLAE---AVAWNGISAGADTFTFSSGDKALLTKSGVDLKAGSEKLSNTKK
Tamavidin 1         ILTQWLLSRSS-EREDLWQSTHVGHDEFSKTKPTKEKIAQAQLL--RRGLKFE----
Tamavidin 2         ILTQWLLSSST-ARGDVWESTLVGNDSFTKTAPTEQQIAHAQLH--CRAPRLK----
Xenavidin           LHTMWLLRSSQEKEQDNWTGTRVGGNTFTRLSKKKIRKE------------------
S. virginiae        INTQWLLTSGT-TEANAWKSTLVGHDTFTKVKPSAASIDAAKKAGINNGNPLDAPQQ
S. katrae           INTQWLLTSGT-TAADSWKSTLVGHDTFTKVKPSAASIDAAKKAGVNNGNPLDAVQQ
S. flavotricini     IVTQWLLSYGT-TPADQWKSTVLGHDEFTRVKPSAADIEKARRLGVTTANPQASEGE
S. hygroscopicus    ILTTWLLSRST-ATAEAWESSVVGQDVFTRQKPTSEDVDRALRSGRPVSHPMPS---
```

b

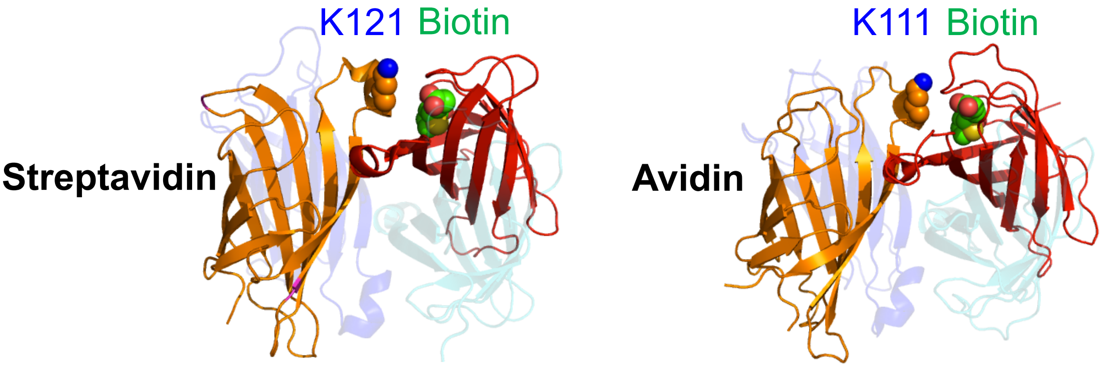

Fig. S2 (related to Fig. 2). Conservation of biotin-proximal lysine in the avidin family. (a) Amino acid sequence alignment of streptavidin-related tetramers, including well-characterized avidins (streptavidin, avidin, neutralite avidin, bradavidin I, tamavidin 1 & 2, and xenavidin) plus four other variants from the *Streptomyces* genus. Fully conserved residues are colored in red. The equivalent residue to streptavidin's K121 is boxed and is colored blue if Lys is conserved. Dimeric avidins such as rhizavidin were not included because they do not possess the relevant protein interface. (b) Structural conservation of the proximity between Lys in subunit 1 (orange) and biotin in subunit 2 (red) of streptavidin (PDB 3RY2) and avidin (PDB 1AVD).

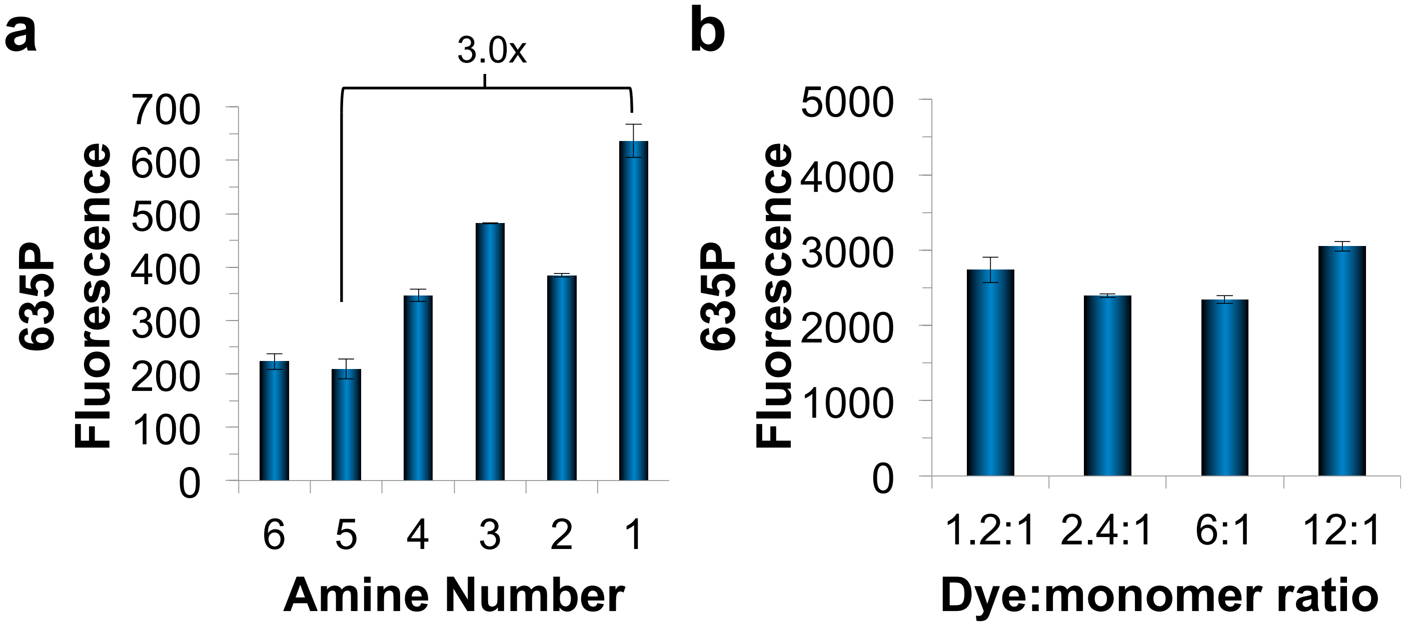

Fig. S3 (related to Fig. 3). Brightness of streptavidin-dye conjugates. (a) The relationship between fluorescence brightness and amine number was maintained at low protein concentration. Fluorescent brightness (in arbitrary units) of amine mutation series conjugated with 635P-NHS, tested with 0.1 µM protein (mean of triplicate ± 1 s.d.). (b) Fluorescent brightness of WT streptavidin after incubation at the indicated dye:monomer ratios with 635P-NHS. Measurement of fluorescence (in arbitrary units) for protein-dye conjugates (mean of triplicate ±1 s.d.).

a

WT	MAEAGITGTWYNQLGSTFIVTAGADGALTGTYESAVGNAE
Flavidin	MAEAGITGTWYNQLGSTFIVTAGADGALTGTYESAVGNAE
WT	SRYVLTGRYDSAPATDGSGTALGWTVAWKNNYRNAHSATT
Flavidin	SRYVLTGRYDSAPATDGSGTALGWTVAW**R**NNYRNAHSATT
WT	WSGQYVGGAEARINTQWLLTSGTTEANAWKSTLVGHDTFT
Flavidin	WSGQYVGGAEARINTQWLLTSGTTEANAW**R**STLVGHDTFT
WT	KVKPSAASEEEEEE
Flavidin	**R**V**R**PSAASEEEEEE

b

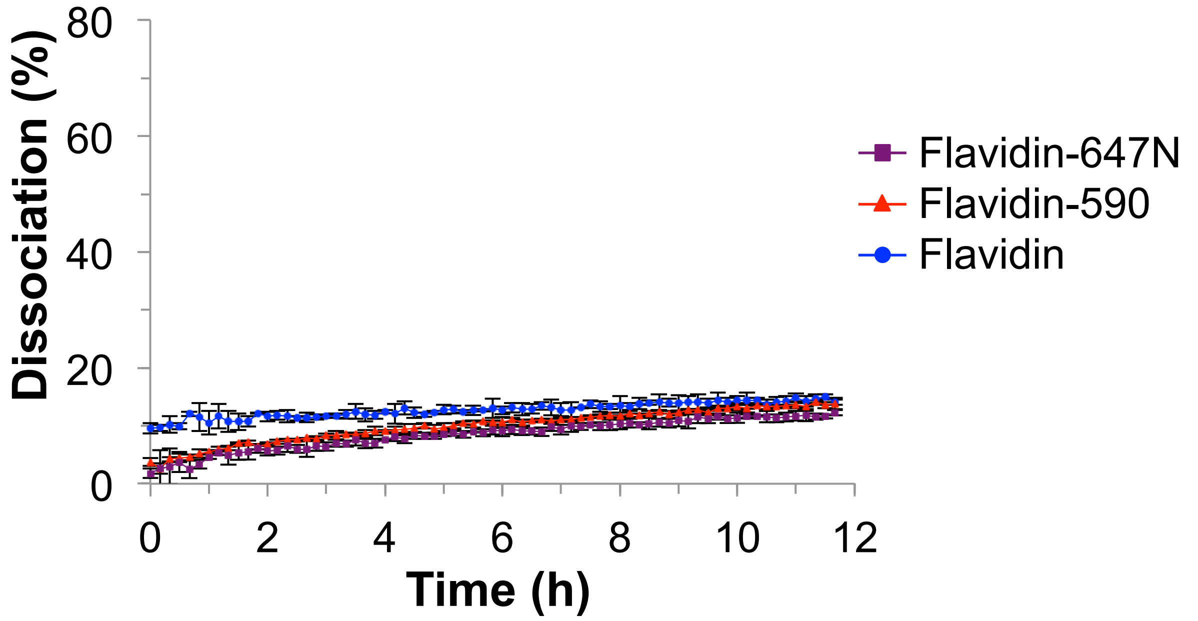

Fig. S4 (related to Fig. 4). Flavidin sequence and ligand dissociation. (a) Amino acid sequences of WT core streptavidin and Flavidin. Mutated residues in Flavidin are marked in red. There is a hexaglutamate tag at each C-terminus for purification. (b) Flavidin ligand dissociation rate was not affected by labeling with alternative dyes. Biotin-4-fluorescein dissociation rates for Flavidin unmodified or after conjugation with Atto647N-NHS or Atto590-NHS (mean of triplicate ± 1 s.d.).

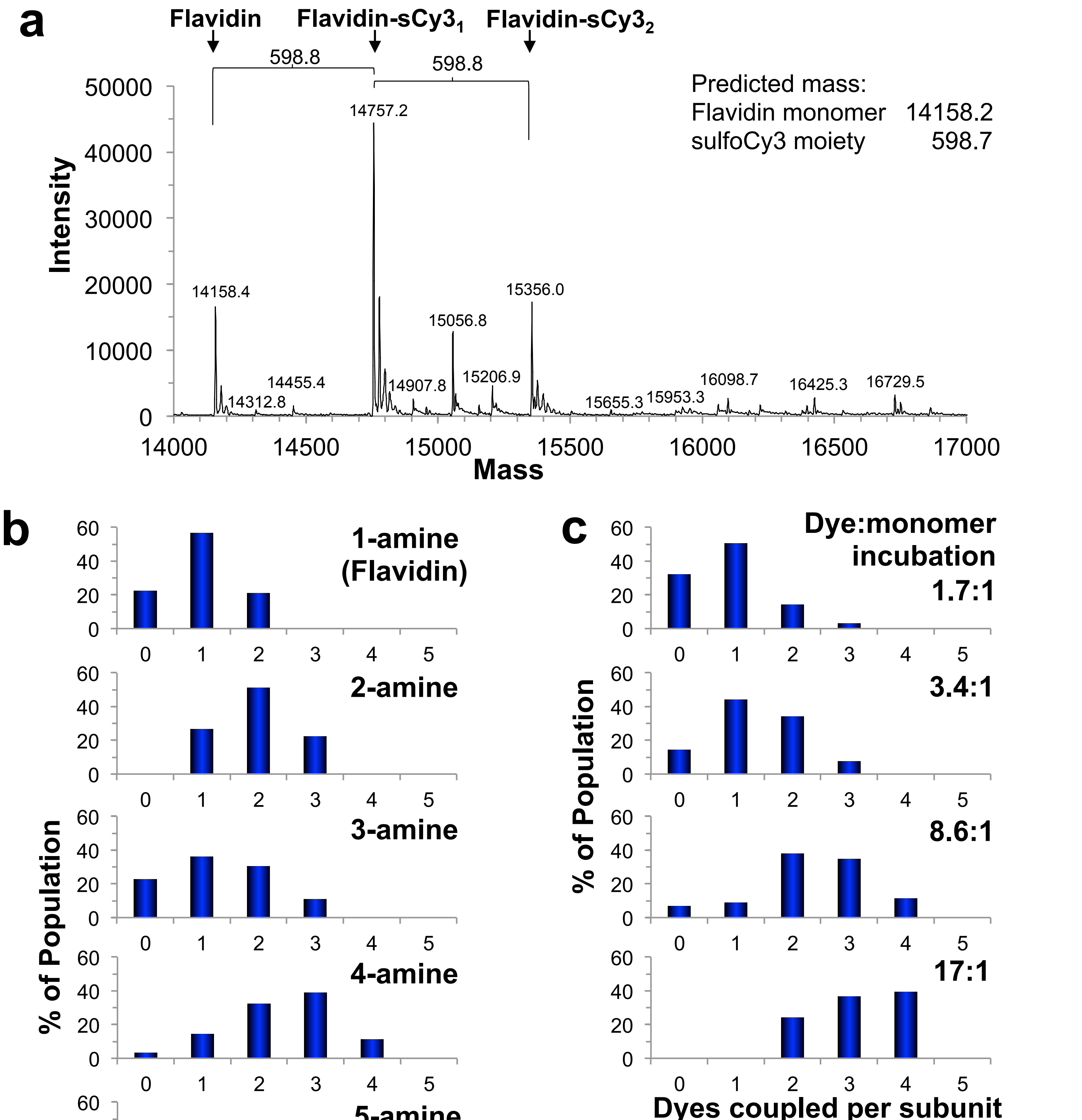

Fig. S5 (related to Fig. 5). Mass spectrometry of labeled streptavidins. (a) Deconvoluted mass spectrum of sulfoCy3-NHS-labeled Flavidin, with monomers containing 0, 1 or 2 dyes indicated. (b) Quantification by MS of sulfoCy3 dyes attached per monomer for the amine series of streptavidins. (c) Quantification by MS of sulfoCy3 dyes attached per monomer for WT streptavidin incubated at the indicated dye:monomer ratio.

Table S1: Oligonucleotides used in this work. Related to STAR Methods section.

Purpose	Oligonucleotide	Oligonucleotide Sequence (5' to 3')
Introduce K121R mutation by QuikChange	SA-K121R-For	GCTAACGCGTGGCGATCCACCCTGGTTGG
…	SA-K121R-Rev	CCAACCAGGGTGGATCGCCACGCGTTAGC
Introduce K121E mutation by QuikChange	SA-K121E-For	GCTAACGCGTGGGAATCCACCCTGGTTGG
…	SA-K121E-Rev	CCAACCAGGGTGGATTCCCACGCGTTAGC
Introduce K121Q mutation by QuikChange	SA-K121Q-For	GCTAACGCGTGGCAATCCACCCTGGTTGG
…	SA-K121Q-Rev	CCAACCAGGGTGGATTGCCACGCGTTAGC
Introduce K121A mutation by QuikChange	SA-K121A-For	GCTAACGCGTGGGCATCCACCCTGGTTGG
…	SA-K121A-Rev	CCAACCAGGGTGGATGCCCACGCGTTAGC
Introduce N82K mutation by Gibson Assembly	SA-N82K-Gib-For	CCGTTGCTTGGAAAAACAAATACCGTAACG CTCACTCCGCTACCACC
…	AmpB	GATCGTTGTCAGAAGTAAGTTGGCC
…	SA-N82K-Gib-Rev	GTTTTTCCAAGCAACGGTCCAACC
…	AmpA	GGCCAACTTACTTCTGACAACGATC
Introduce R103K mutation by Gibson Assembly	SA-R103K-Gib-For	CAGTACGTTGGTGGTGCTGAAGCTAAAATC AACACCCAGTGGTTGTTGACC
…	AmpB	GATCGTTGTCAGAAGTAAGTTGGCC
…	SA-R103K-Giv-Rev	ACCACCAACGTACTGGCCAGACC
…	AmpA	GGCCAACTTACTTCTGACAACGATC
Introduce K80R mutation by Gibson Assembly	SA-K80R-Gib-For	TCTGGGTTGGACCGTTGCTTGGCGCAACAA CTACCGTAACGCTCAC
…	AmpB	GATCGTTGTCAGAAGTAAGTTGGCC
…	SA-K80R-Gib-Rev	CCAAGCAACGGTCCAACCCAGAG
…	AmpA	GGCCAACTTACTTCTGACAACGATC
Introduce K132R and K134R mutations by Gibson Assembly	SA-K132RK134R-For	GGTCACGACACCTTCACCCGTGTTCGTCCG TCCGCTGCTTCCG
…	AmpB	GATCGTTGTCAGAAGTAAGTTGGCC
…	SA-K132RK134R-Rev	GGTGAAGGTGTCGTGACCAACC
…	AmpA	GGCCAACTTACTTCTGACAACGATC
